# DNA Methylation in the Human Cerebral Cortex Is Dynamically Regulated throughout the Life Span and Involves Differentiated Neurons

**DOI:** 10.1371/journal.pone.0000895

**Published:** 2007-09-19

**Authors:** Kimberly D. Siegmund, Caroline M. Connor, Mihaela Campan, Tiffany I. Long, Daniel J. Weisenberger, Detlev Biniszkiewicz, Rudolf Jaenisch, Peter W. Laird, Schahram Akbarian

**Affiliations:** 1 Department of Preventive Medicine, Keck School of Medicine, University of Southern California, Los Angeles, California, United States of America; 2 Program in Neurobiology, Graduate School of Biomedical Sciences, University of Massachusetts Medical School, Worcester, Massachusetts, United States of America; 3 Department of Psychiatry, University of Massachusetts Medical School, Worcester, Massachusetts, United States of America; 4 Department of Biochemistry and Molecular Biology, Keck School of Medicine, University of Southern California, Los Angeles, California, United States of America; 5 The Whitehead Institute for Biomedical Research, Cambridge, Massachusetts, United States of America; University of Washington, United States of America

## Abstract

The role of DNA cytosine methylation, an epigenetic regulator of chromatin structure and function, during normal and pathological brain development and aging remains unclear. Here, we examined by MethyLight PCR the DNA methylation status at 50 loci, encompassing primarily 5′ CpG islands of genes related to CNS growth and development, in temporal neocortex of 125 subjects ranging in age from 17 weeks of gestation to 104 years old. Two psychiatric disease cohorts—defined by chronic neurodegeneration (Alzheimer's) or lack thereof (schizophrenia)—were included. A robust and progressive rise in DNA methylation levels across the lifespan was observed for 8/50 loci (*GABRA2, GAD1, HOXA1, NEUROD1, NEUROD2, PGR, STK11, SYK*) typically in conjunction with declining levels of the corresponding mRNAs. Another 16 loci were defined by a sharp rise in DNA methylation levels within the first few months or years after birth. Disease-associated changes were limited to 2/50 loci in the Alzheimer's cohort, which appeared to reflect an acceleration of the age-related change in normal brain. Additionally, methylation studies on sorted nuclei provided evidence for bidirectional methylation events in cortical neurons during the transition from childhood to advanced age, as reflected by significant increases at 3, and a decrease at 1 of 10 loci. Furthermore, the DNMT3a *de novo* DNA methyl-transferase was expressed across all ages, including a subset of neurons residing in layers III and V of the mature cortex. Therefore, DNA methylation is dynamically regulated in the human cerebral cortex throughout the lifespan, involves differentiated neurons, and affects a substantial portion of genes predominantly by an age-related increase.

## Introduction

Epigenetic modification of chromatin, including DNA methylation at the sites of CpG dinucleotides, is a key regulator of gene expression, growth and differentiation in virtually all tissues, including brain [1], [2], [3], [4]. Dysregulated DNA methylation, or methyl-CpG-dependent chromatin remodeling, is thought to underlie *ICF* syndrome (*I*mmunodeficiency, *C*entromere instability and *F*acial anomalies), Rett's disorder and other mental retardation syndromes [5], [6]. Furthermore, changes in methylation status at selected genomic loci may affect social cognition [7], learning and memory [8] and stress-related behaviors [9] and is believed to contribute to dysregulated gene expression in a range of adult-onset neuropsychiatric disorders, including autism, schizophrenia, depression and Alzheimer's disease [10], [11], [12], [13], [14]. Finally, there is strong evidence that aberrant methylation of tumor suppressor genes contributes to the molecular pathology of a subset of astrogliomas and other types of brain cancers [15], [16].

However, despite its clinical importance, the regulation of DNA cytosine methylation, particularly in the human brain, remains poorly understood. To date, there are no comprehensive studies which have monitored methylation at multiple loci during the course of brain development and aging, or in chronic psychiatric disease. Furthermore, all previous studies of DNA methylation in human or animal brain utilized tissue homogenates comprised of a highly heterogeneous mixture of neurons and glia [8], [9], [12], [17], or examined DNA methylation in subfractions of chromatin defined by site-specific histone modifications [18] and therefore it remains to be determined whether or not DNA methylation is dynamically regulated in terminally differentiated neurons.

Given this background, the present study was undertaken to provide a first insight into the dynamics of DNA methylation in the human cerebral cortex. Altogether, we examined 50 loci, mostly CpG islands within the 5′ end of genes, during the course of development, maturation and aging. Additionally, we assessed the methylation status for these same loci in Alzheimer's disease and schizophrenia; the former condition is characterized by chronic neurodegeneration and the latter by widespread transcriptional and metabolic perturbations [19], [20], [21] in the absence of large scale loss of neurons. While disease-associated alterations were limited to 2/50 sequences in the Alzheimer's cohort of the present study, the majority of genomic loci, including genes implicated in neural development and CNS tumors, showed a striking age-associated increase in methylated CpGs. Furthermore, we show that DNA methylation is dynamically regulated in differentiated neurons during the transition from childhood to advanced age. Collectively, our results suggest that DNA methylation in the human cerebral cortex, including its neuronal constituents, is dynamically regulated across the full lifespan and potentially affects a substantial portion of the genome.

## Results

### Four types of age-related DNA methylation profiles in the human cerebral cortex

**Figure 1:**
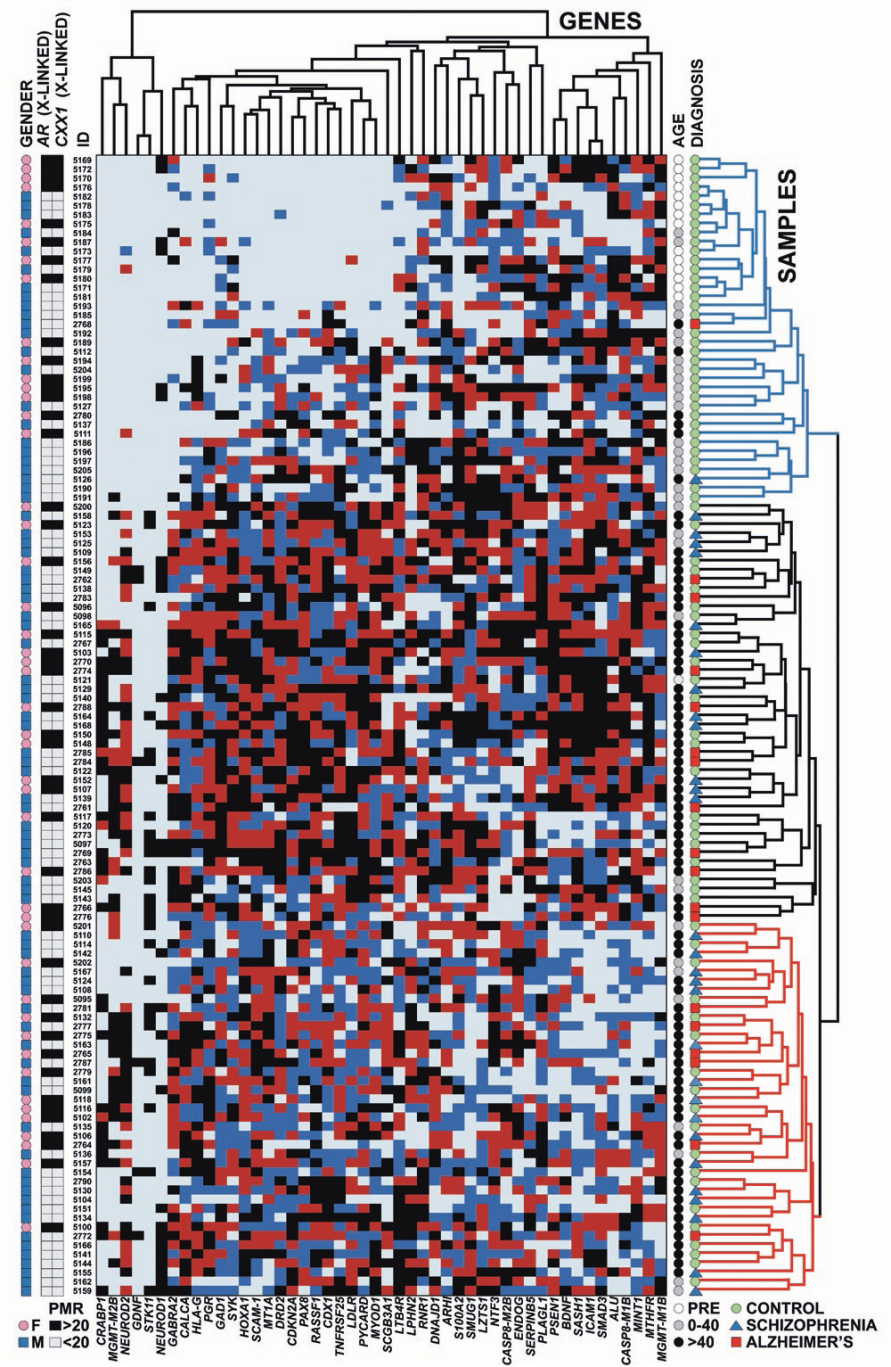
DNA methylation changes at 50 loci in temporal neocortex across the lifespan. Two dimensional hierarchical cluster analysis using Manhattan distance and average linkage (N = 48 regions, 125 subjects). DNA (rostro-lateral temporal neocortex) was extracted and analyzed by MethyLight for the genes indicted as described (see Methods). Each gene is grouped into quartiles (Dark Light Blue = 1 low, Dark Blue = 2 medium-low, Red = 3 medium high, Black = 4 high extent of methylation). The larger the number of samples with no detectable methylation, the fewer the number of observations coded dark blue and red. Gender (Blue squares = Male, Pink circles = Female), Age (White circles = Prenatal (PRE); Gray circles = 0–40 years old; Black circles = older than 40 years), and Diagnosis (Green circles = controls; Blue triangles = Schizophrenia cases; Red squares = Alzheimer's cases) are indicated with symbols explained below each variable. *AR* and *FAM127A* are two additional X-linked genes with DNA methylation occurring on the inactive X-chromosome in females, and are dichotomized at a PMR value of 20, as indicated. PMR = Percent of Methylated Reference [23], [55]. Three major sample clusters are indicated on the right hand side in blue (consisting mostly of prenatal and young adult samples), black, consisting mostly of subjects over 40 years old with high density CpG island methylation, and red, mostly subjects over 40 years old with lower density CpG island methylation.

Using a real-time PCR-based technique called MethyLight [22], [23], we analyzed DNA methylation at 50 loci, most of them representing promoter CpG islands of genes expressed in the cerebral cortex; a portion of these genes is also implicated in cancer biology (Table S1 and Table S2). Most of the cancer-related genes included in this study show aberrant methylation in various types of neoplasia, including CNS tumors (Table S1), and hence we were interested to monitor potential methylation changes within these genes during the course of normal brain development and aging. Other genes included in this study play a role in the molecular pathology of some cases diagnosed with schizophrenia and other psychiatric illness (e.g., *BDNF, DRD2, GABRA2, GAD1, HOXA1, NTF3*) or are linked to chronic neurodegeneration (*LDLR, PSEN1, S100A2*). We screened altogether 125 pre- and postnatal, child and adult samples of rostro-lateral temporal cortex, yielding 7,500 quantitative measurements (**Fig. 1**). Two of the CpG islands (*AR* and *FAM127A*) are from X-linked genes, which become methylated only on the inactive X-chromosome in females, and were included as internal controls to validate the DNA methylation measurements (**Fig. 1**). For 124 (out of 125) samples, *AR* and *FAM127A* methylation levels were in agreement with the gender information provided by the brain bank, and for the remaining one case, the discrepancy was resolved upon re-review of the chart on file with the bank. Therefore, all of the female postmortem samples, but none of the males, showed substantial DNA methylation of both of these X-linked genes, as expected.

**Figure 2:**
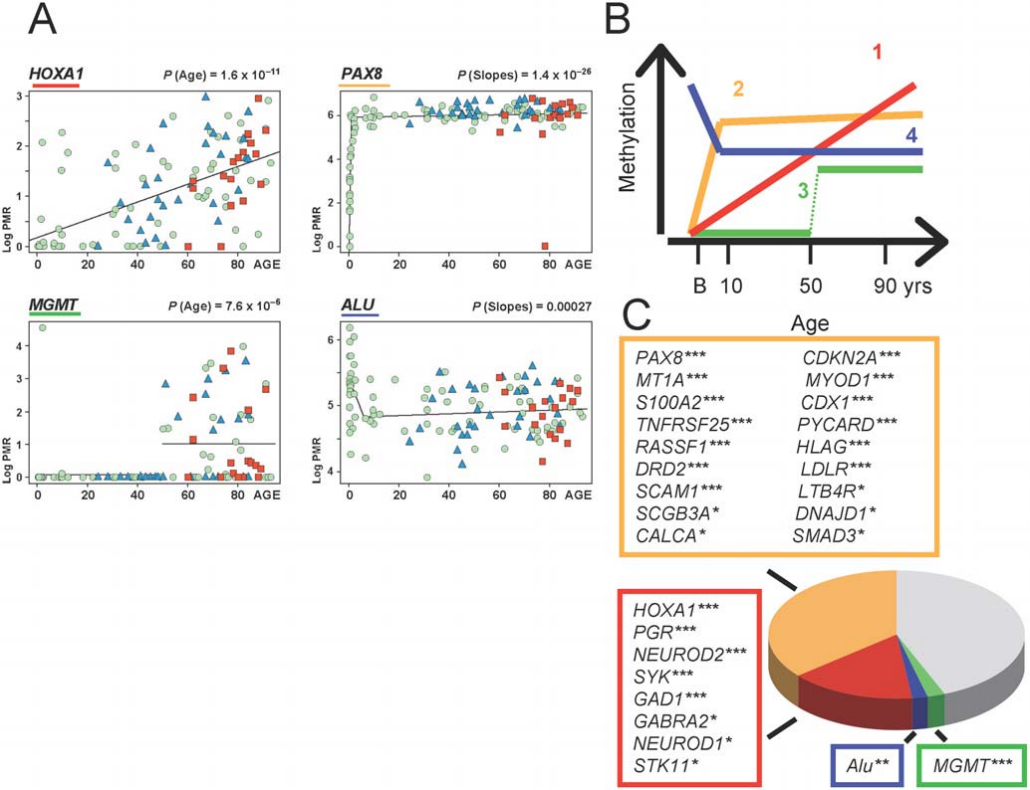
Four developmental profiles for cortical DNA methylation. (A) Associations of log-transformed PMR values (ln(PMR+1); y-axis) with age (x-axis) for several representative genes. Trends are studied using linear regression. *HOXA1* shows a linear association. *MGMT*-M2B shows a non-linear shift, with the *P*-value referring to a *T*-test of the difference in mean methylation value for subjects under or over 50 years of age. For the biphasic linear trends of *PAX8* and the *ALU* sequence *ALU-M1B* (Table S2) , the *P*-values refer to a test of change of slope. Green dots = controls, blue triangles = schizophrenia and red squares = Alzheimer's subjects. (B) Schematic summary of the four different types (1–4, see text for details) of developmental methylation profiles in human temporal cortex across the lifespan (x-axis, B = birth), as illustrated by the representative examples in (A). (C) Listings and proportion of gene loci (total = 48; excluding *AR* and *FAM127A* which showed gender-specific methylation) that show significant age-dependent methylation changes: colors refer to the scheme in (B). Gray sector refers to the subset of loci without a significant age effect. N = 125 subjects. All p-values are adjusted for diagnosis; *** p<0.0001, ** p<0.001, * p<0.05.

Unsupervised hierarchical clustering of the remaining 48 gene loci, excluding the X-linked genes, revealed a strong age association (**Fig. 1**). All prenatal samples were contained in a single cluster, shown at the top of **Fig. 1** (blue cluster). The subjects over 40 years of age were divided into two other major clusters with either moderate amounts (red cluster at bottom) or high density of CpG island methylation (black cluster in middle). The majority of loci showed a statistically significant increase of DNA methylation associated with age, adjusted for diagnosis, but the chronology of these age-associated changes varied remarkably among different loci. Altogether, four different types of age-related methylation changes were discernible: (1) Eight of the 50 gene loci showed a linear increase of DNA methylation with age, as exemplified by *HOXA1* (**Fig. 2** and Figure S1). (2) Half of the genomic loci showed a statistically significant biphasic age distribution (Figure S1). Among these, 18 genes revealed a sharp shift in slope at some time-point within the first decade of life, mostly in the newborn period, as indicated by *PAX8* (**Fig. 2** and Figure S1). (3) One locus (*MGMT*) showed a highly unusual, non-linear stochastic accumulation of hypermethylation starting at about age 50 (**Fig. 2** and Figure S1). The stochastic nature of this hypermethylation event is of particular interest, since variation in *MGMT* CpG island hypermethylation in gliomas is associated with clinical response to alkylating agents [24]. Interestingly, the incidence of malignant gliomas peaks around age 40–50 [25], [26], which is the same age period when *MGMT* hypermethylation emerged in the samples of the present study. (4) Finally, in striking contrast to the age-related progressive increase in DNA methylation at single copy genes (described above under type 1,2,3), *ALU* and other repetitive elements either showed a significant *decrease* in DNA methylation during the first decade of life, followed by relatively little change during subsequent maturation and aging (1/5 repetitive sequences) (**Fig. 2**), or showed relatively little change across the lifespan (4/5 repetitive sequences) (Figure S1). For the majority of loci showing an age-related increase in DNA methylation, the effect was extremely robust (p<0.0001) (**Fig. 2** and Figure S1).

While it was beyond the scope of this study to assess the relationship between the observed age-related methylation pattern and corresponding changes in gene expression for all loci, we hypothesized that genes showing a linear and robustly progressive increase in methylation throughout the lifespan (“type 1” genes, see above and **Fig. 2**) would show a decline in mRNA levels at advanced age. To examine this, we profiled temporal cortex mRNA levels by qRT-PCR for 4 of the “type 1” genes listed in **Fig. 2** (*SYK, NEUROD2, GABRA2, GAD1*) in a cohort of six child brains (range: 1.3–11.5 years) and 11 adults (range: 32–87 years), carefully matched for RNA quality (see Methods) and normalized to *18S* rRNA levels (data not shown). Indeed, all four genes showed an inverse correlation between mRNA levels and age, to a moderate degree (R^2^ = 0.29–0.42 for *SYK*, *NEUROD2*, *GAD1* and *GABRA2*). In contrast, mRNA levels of *B2M* and *GUSB*, two housekeeping genes commonly used to assess RNA quality in human postmortem specimens [27], and of *MGMT*–a gene with a highly unusual age-related methylation profile (**Fig. 2**)–did not show a correlation with age (R^2^ = 0.02 for *MGMT*, and <0.002 for *B2M* and *GUSB*). Therefore, the age-related decline in mRNA levels observed for a subset of the “type 1” genes is not explained by generalized RNA deficit or decay in the older specimens. We conclude that the progressive, age-related increases in DNA methylation at the 5′ sequences of these genes could contribute to the observed age-related decline in corresponding mRNA levels.

### Disease-associated alterations

**Figure 3:**
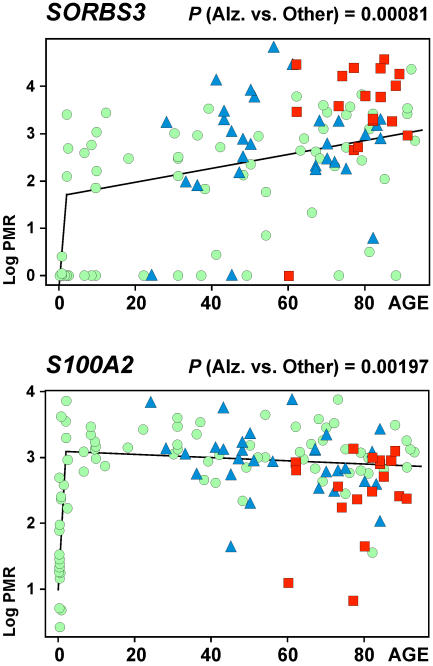
Acceleration of age-associated DNA methylation changes in Alzheimer's disease. Scatter plots showing age-associated methylation changes for *SORBS3* and *S100A2* across all ages. N = 125 subjects, including Alzheimer subjects (red squares), schizophrenics (blue triangles) and controls (green dots). P-values refer to T-tests for comparison of Alzheimer subjects versus all other subjects older than 60 years. One outlier (ID 2763, 104 years old, Table S4) was omitted from the age-associated analyses.

Taking a false discovery rate into account, Alzheimer's cases showed a statistically significant difference in DNA methylation for *SORBS3* and *S100A2*. In both cases, the Alzheimer patients tend to show an acceleration of the age-associated changes in DNA methylation (**Fig. 3**). *SORBS3* (also known as *vinexin, SCAM-1* or *SH3D4*), which encodes a cell adhesion molecule expressed in neurons and glia [28], becomes progressively more likely to be methylated with age, and is methylated to a greater degree in Alzheimer patients (median PMR = 38.5, N = 18) than in all other cases (schizophrenia, controls) older than 60 years (median PMR = 16.9, N = 39, *P* = 0.00081). *S100A2*, a member of the S100 family of calcium binding proteins, displays a complex chronology, with a rapid prenatal increase, followed by an infrequent stochastic decrease in DNA methylation later in life, particularly among Alzheimer patients (median PMR = 12.9, N = 18) versus all other subjects older than 60 years (median PMR = 20.5, N = 39, *P* = 0.00197). The age- and disease-associated loss of *S100A2* DNA methylation in Alzheimer's disease is consistent with the observation of S100A2 protein in corpora amylacea, or polyglucosan bodies, which accumulate in aging human brains [29]. Therefore, the significant DNA methylation changes in Alzheimer's disease, including the decrease of *S100A2* and increase in *SORBS3* CpG methylation, appear to represent accelerations of the normal, age-associated DNA methylation changes in these genes. Notably, previous in vitro studies provided evidence that DNA methylation is involved in transcriptional regulation of *PSEN1* [10] an Alzheimer's disease-associated gene and regulator of amyloid precursor protein and Notch signaling pathways [30]. However, *PSEN1* showed only very low levels of methylation in our samples, and we did not find age- or disease-associated changes (Table S3, part a). This lack of consistent change in PSEN1 methylation in diseased or aging tissue, however, may not be surprising given that this gene exhibits significant variability in interindividual methylation, according to a study in male germ cells [31].

The methylation of *PAX8*, a gene encoding a paired box-containing transcription factor important for CNS and thyroid development [32], was higher in schizophrenics than in controls (P = 0.0025) (Table S3, part b), but this was not considered statistically significant after adjusting for multiple comparisons by controlling the false discovery rate (set at 0.05) [33]. We conclude that schizophrenia is not accompanied by consistent DNA methylation changes at the 50 gene loci included in this study.

### The *de novo* DNA methyltransferase, DNMT3a, is expressed in developing and aging cerebral cortex

**Figure 4:**
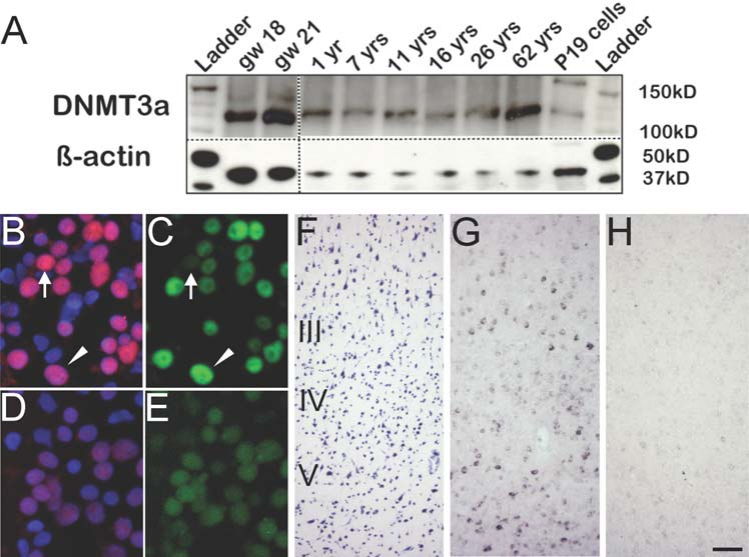
Developmental and cellular expression pattern of DNMT3a in the cerebral cortex. (A) Representative immunoblotting of temporal cortex homogenates with anti-DNMT3a antibody (top) and β-actin as loading control (bottom). Left, fetal samples (gw = gestational week); right, child and adult brains (yrs = years) and, as positive control, murine embryonic carcinoma, “P19” cells. Notice expression of DNMT3a—indicated by a ∼120 kDa band—across all ages. (B, C) Digitized images of temporal cortex nuclei from 1 year old infant, processed for DNMT3a (red) and NeuN (green) immunoreactivity and counterstained with DAPI. Notice numerous neuronal nuclei expressing both markers, including representative example marked by arrowhead. Occasional non-neuronal DNMT3a+ nucleus is marked by arrow. (D,E) show weak background staining and formalin fixation-related autofluorescence in negative controls processed without primary antibodies. (F–H) Images from layers III, IV and V of parallel sections from adult temporal cortex, stained for Nissl (F) or hybridized with digoxigenin-labeled DNMT3a antisense (G) or sense riboprobe (H). Notice in (G) robust expression of DNMT3a mRNA in a subset of layer III and V neurons. Images in B–E taken at with 20× objective. Bar (F–H) in H = 100 µm.

The DNA methylation data described above strongly suggest that DNA methylation events in the cerebral cortex are ongoing across a wide age range, extending beyond the developmental period and continuing into old age. If this hypothesis is correct, then one would expect expression of the *de novo* DNA methyltransferase enzymes, DNMT3a and/or DNMT3b [34], [35], at all ages. In mouse cerebral cortex, Dnmt3a expression remains detectable in adults, albeit at lower levels than observed during earlier periods of postnatal development; in contrast, Dnmt3b is found in murine CNS only during a narrow period of prenatal development [36]. To find out when DNMT3a protein is expressed in the human cerebral cortex, we employed immunoblotting on cortical homogenates from fetal, child and adult samples. We observed, across all ages, an immunoreactive band of approximately 120 kDa, corresponding to full-length form of DNMT3a [37], [38] (**Fig. 4A**). Immunolabeling of intact nuclei from child and adult cortex revealed that the bulk of the DNMT3a-like immunoreactivity is derived from neuronal nuclei (**Fig. 4B–E**). Expression of DNMT3a in neurons was confirmed by in situ hybridization studies with full length DNMT3a cRNA (**Fig. 4F–H**); a subset of neurons, including some with pyramidal neuron-like morphology (**Fig. 4G**) residing in layers III and V of the adult cortex expressed DNMT3a mRNA. We conclude that DNMT3a in human cerebral cortex is expressed primarily in neurons, which is in agreement with similar findings in mice [36] and, furthermore, is expressed across the lifespan from the 2^nd^ trimester of pregnancy through old age.

### Age-related DNA methylation changes in nuclei of differentiated neurons

**Figure 5:**
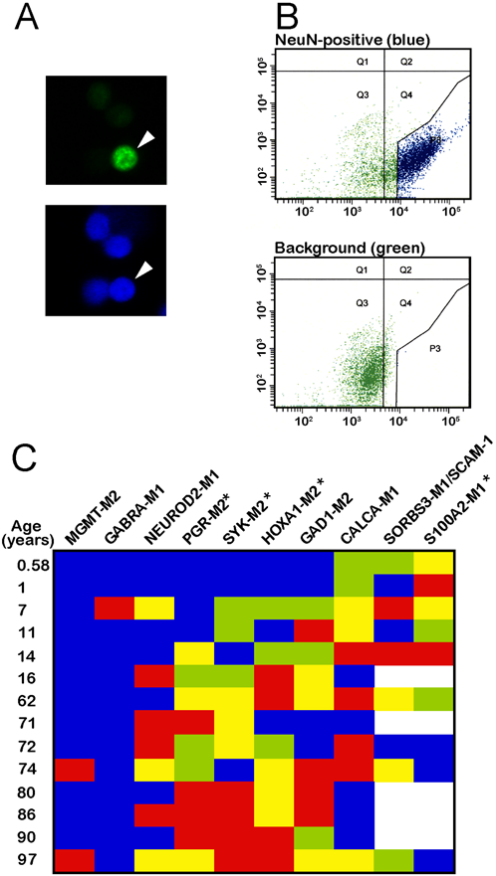
Age-related DNA methylation changes in differentiated neurons. (A) Examples of nuclei stained with anti-NeuN (green) and counterstained with DAPI; arrowhead marks double-labeled cell. Notice absence of detectable background and autofluorescence in these samples that were processed without prior fixation (B) Representative FACS of unfixed NeuN labeled material similar to the one shown in (A) (top) and of negative control (bottom); blue dots mark sorted neuronal nuclei (NeuN+). (C) Heatmap showing methylation levels of neuronal DNA isolates for 10 different genes across the lifespan (range: 0.6–97 years). Each gene is grouped into quartiles (blue = 1 low, green = 2 medium-low, yellow = 3 medium high, red = 4 high extent of methylation). The larger the number of samples with no detectable methylation, the fewer the number of observations coded green and yellow. White space = no data. * = p<0.05 , Mann-Whitney U permutation based on comparison of immature/young (0.6–16 years) to old (62+years) samples. Notice that neuronal DNA from advanced age group shows significant increase in DNA methylation for 3/10 gene loci (*PGR, SYK, HOXA1*), but decreased levels at *S100A2* locus.

Notably, studies in rat and mouse identified a number of stimuli or environmental conditions that alter expression of selected mRNAs in immature, or mature brain, in conjunction with–often bidirectional–changes in CpG methylation of the corresponding promoters [8], [9], [39], [40]. The conclusion drawn by these studies, either explicitly or implicitly, is that neuronal gene expression is subject to epigenetic regulation. However, most CNS tissues, including cerebral cortex, are comprised of a highly heterogeneous mixture of various dividing and postmitotic cell populations, which are likely to show important differences in the methyl-CpG patterning of their genomes. This uncertainty regarding the cellular specificity of any DNA methylation signal obtained from brain homogenates places limitations on the interpretation of the age-related changes in methylation as described above. Nonetheless, the presence of DNMT3a in cortical neurons across a wide age range (**Fig 4**), in conjunction with the robust, age-related methylation changes at >50% of the gene loci (**Fig. 1** **,** **2**), strongly suggests an ongoing modification of the neuronal DNA long after the exit from the cell cycle, which in primate cerebral cortex occurs during fetal mid-development [41]. To find out whether DNA methylation is dynamically regulated in postmitotic neurons and to rule out the potential confound of changes in glia numbers during the course of development [42], [43], we isolated nuclei of differentiated neurons from child and adolescent (1–16 years) and aged (>60 years) cortex using NeuN immunolabeling in conjunction with fluorescence-activated cell sorting (FACS) (**Fig. 5A,B**). Then, the methylation levels for 10 gene loci were analyzed in the neuronal DNA by MethyLight PCR. When compared to child and adolescent specimens, DNA from aged neuronal nuclei showed a significant increase in methylation at 3/10 loci (*HOXA1, PGR, SYK*), and a significant decrease in 1/10 loci (*S100A2*) (**Fig. 5C**). Therefore, during the transition from childhood to old age, differentiated cortical neurons undergo bidirectional changes in DNA methylation.

## Discussion

The present study examined DNA methylation changes for 50 genomic loci during the course of development, maturation and aging of the human cerebral cortex. The majority of loci showed significant age effects: eight loci showed a progressive increase in methylation that continued across the entire lifespan and another 18 loci were defined by a sharp rise within the first months or years after birth. We present direct evidence that, for a subset of loci, genomic DNA from differentiated cortical neurons undergoes methylation changes during the course of maturation and aging. In addition, one locus, *MGMT*, showed a stochastic accumulation in methylation starting around age 50, with potential implications for the tumor biology of astrogliomas, as discussed above. While DNA methylation changes related to development or aging were extremely robust in the present study, disease-associated changes, on the other hand, were surprisingly limited. Schizophrenia, a chronic psychiatric disease condition associated with psychosis and widespread cortical dysfunction in the absence of large-scale loss of neurons [44], [45], [46], was not associated with significant methylation changes in the present study. On the other hand, cases diagnosed with Alzheimer's disease, which is defined by a neurodegenerative process in cerebral cortex and other brain regions, showed significant methylation changes in 2/50 loci. One locus (*S100A2*), which is methylated in neurons (**Fig. 5C**) was significantly less methylated in the DNA from Alzheimer cases compared to age-matched controls (**Fig. 3**), possibly due to large-scale loss of neurons associated with that disease. In addition, methylation of another locus (*SORBS3*) was higher in the Alzheimer samples than in controls. Thus, the DNA methylation alterations in both genes appear to reflect an enhancement, or acceleration, of the age-associated changes that we observed in normal brain (**Fig. 3**).

It is noteworthy that the overwhelming majority of loci analyzed in the present study demonstrated age-related *increases* in DNA methylation in cerebral cortex (26/50 loci), and only one gene–*S100A2*–showed a change in the opposite direction; this decrease was even more pronounced in the Alzheimer's cohort. Likewise, in DNA samples derived selectively from differentiated neurons of controls, only *S100A2* showed an age-related loss of methylation, while significant increases were found for 3/10 genes (**Fig. 5C**). Collectively, these findings support the notion that DNA methylation levels progressively increase in the cerebral cortex at many genomic loci during the course of maturation and aging. On the other hand, according to the findings presented here, DNA de-methylation events appear to play a less prominent role. Therefore, it remains to be clarified whether or not there is active de-methylation in brain, and if DNA repair-related mechanisms are involved similar to those recently reported for dividing cells and *Xenopus* oocytes [47]. Additionally, further studies will be required in order to determine whether or not DNA methylation/demethylation in human brain is subject to more acute alterations—on the scale of hours or days—as has been previously demonstrated in cell cultures and animal models [39], [40], [48].

It is important to realize that our study had several limitations, including the focus on one area of the cerebral cortex, i.e. the neocortex of the anterior and lateral temporal lobe. Therefore, additional studies will be necessary to confirm that the developmental DNA methylation changes as observed in this study are a generalized feature operating throughout all areas of the human cerebral cortex. Furthermore, we monitored DNA methylation changes at a limited number of genomic sequences, hence it will be necessary to confirm the findings reported here on a more comprehensive, genome-wide scale. Such studies will be necessary in order to find out (i) whether or not the developmental DNA methylation changes reported here represent a more generalized, age-dependent drift towards increased methylaton levels and (ii) whether or not schizophrenia or Alzheimer's disease are associated with DNA methylation changes affecting wide-spread portions of the genome. Finally, while our study presents some of the first and direct evidence for methylation changes in the DNA of terminally differentiated neurons, our analyses was limited to samples obtained from children and adults, because isolation of neuronal nuclei from fetal specimens via FACS was not feasible for technical reasons. Hence, it remains to be determined whether or not neurons, or various types of glia and other non-neuronal cells, contribute to the observed sharp rise in DNA methylation during the perinatal and early childhood period that was observed at 18/50 loci in this study. These highly dynamic methylation increases postnatally could either be related to the relatively high levels of neuronal DNMT3a methyl-transferase in the immature brain [36] or , alternatively, result from developmental shifts in cell composition of the postnatal cortex, including a rise in the number of oligodendrocytes and other glia-related changes [49]. In light of these findings, it is tempting to speculate that certain nurturing, feeding and other “environmental” conditions could potentially result in sustained DNA methylation and gene expression changes affecting many parts of the genome. Indeed, emerging evidence from animal models is in support of this hypothesis [9], [50]. Based on the results of the present study, we predict that approximately one half of genes encoded in the genome will show age-related DNA methylation changes in the human brain, many of which will directly, or indirectly, affect neuronal gene expression and thus cognition and behavior.

## Materials and Methods

### Human brain tissue

Fresh frozen, postmortem brain tissue from fetuses, newborns and children were obtained through the Brain and Tissue Banks for Developmental Disorders, University of Maryland and University of Miami (NICHD contract # NO1-HD-8-3284). Adult tissue samples were obtained from three brain banks (i) the Center for Neuroscience, University of California at Davis, CA, (ii) the Harvard Brain Tissue Resource Center at McLean Hospital, Belmont, MA, and (iii) the Massachusetts General Hospital, Boston, MA. All brain banks provided tissue to us without personal identifiers, and all collection and written consent procedures (donors or family members) were approved by the institutional review boards of the brain banks' institution. Small blocks of frozen, unfixed tissue were dissected from the developing cortical plate (fetus) or cerebral cortex (children, adults) of the anterior lateral temporal lobe.

Altogether, 17 fetal, 15 child and 93 adult specimens were included in the present study. Among the adult samples, there were 18 cases meeting CERAD criteria of definite Alzheimer's disease and 30 cases meeting DSM-IV based criteria for schizophrenia (Table S4).

### Methylation and mRNA analyses

From all specimens, DNA was extracted from the cortical plate (fetus) or gray matter (children, adults) using a standard procedure, with modifications [51] and analyzed by MethyLight PCR after bisulfite conversion [22], [23], [52] (for primer sequences, see Table S2). In addition, RNA was extracted from cortical gray matter of child and adult samples with the RNeasy Lipid Tissue Mini Kit (Qiagen, Valencia, CA) and treated with DNAse I. RNA quality for all samples was assessed using high-resolution capillary electrophoresis on the Agilent Bioanalyzer 2100 (Agilent Technologies, Palo Alto, CA). Samples with a RIN<4.0 were discarded [27]. RNA was reverse-transcribed and amplified with TaqMan One-Step RT-PCR Master Mix Reagent in 7500 Real Time PCR System machine (Applied Biosystems, Foster City, CA, U.S.A.), in conjunction with unlabeled primers and SYBR Green (*GAD1*) or FAM-labeled primer sets purchased from Applied Biosystems (all other genes). Quantifications were performed by positioning the cycle threshold within the linear range of amplification curve. Each value of mRNA was calculated with the equation V = (1+E)^Ct^ (E: amplification efficiency) and normalized to 18S ribosomal RNA.

### DNMT3a expression studies

For western blotting, 100 mg aliquots of cortical tissue were homogenized in 1× Laemmli buffer for SDS-PAGE, then processed for anti-DNMT3a immunoreactivity (rabbit polyclonal, Abcam Inc., Cambridge, MA) at a final dilution of 1:250; or, for loading control, mouse anti-β-actin (Sigma, St. Louis, MO), final dilution 1∶10,000.

To extract nuclei for DNMT3a-like immunolabeling, cortical tissue was homogenized in 2mL 1× RSB buffer (100 mM NaCl, 30mM MgCl_2_, 100 mM Tris-HCl, pH 7.5) with 1% NP-40, mixed with 8 mL 1× RSB, and centrifuged in a swing-bucket rotor at 1000×*g* for 10 min at 4°C. Subsequently, the pellet was dissolved in 4 mL 4% phosphate-buffered paraformaldehyde (PFA) and incubated for 10 min at room temperature. This homogenate was layered onto a 30% sucrose cushion, centrifuged, and the resulting pellet dissolved in 0.1% Triton X-100/0.32M sucrose/5 mM CaCl_2_/0.1 mM EDTA/10 mM Tris-HCl, pH 8.0, mixed with 1 mL 1.8 M sucrose, and centrifuged at 1500×*g* at 4°C for 10 min on a 1 mL 1.2 M sucrose cushion. Nuclei pellets were dissolved in 1× PBS, and then dried on glass slides and blocked with 1×PBS/10% normal goat serum/0.2% Triton X-100 for 1 hour. For double immunolabeling, primary antibodies (anti-DNMT3a and anti-NeuN as a neuron-specific marker [53], [54]) were labeled with the Zenon Alexa Fluor 594 Rabbit IgG Labeling Kit or the Enhanced Zenon® (Alexa 488) Mouse IgG Labeling Kit (Invitrogen, Carlsbad, CA) and applied at 1∶500 final dilution to the slides for 4 hrs. Following incubation, slides were rinsed repeatedly, incubated in tyramide solution according to the manufacturer's instructions, washed, counterstained with DAPI and coverslipped.

To prepare for in situ hybridization histochemistry, tissue blocks were allowed to thaw, then immersion-fixed with 4% phosphate-buffered PFA for up to 4 days, and then cryoprotected in 30% phosphate-buffered sucrose; 20 µm sections were cut on a cryostat, mounted on glass slides, and stored at −80°C until use. Sense and antisense digoxigenin (DIG)-labeled DNMT3a cRNA probes were generated from full-length human DNMT3a cDNA (Genbank BC043617) in the presence of DIG-11-UTP (Roche Applied Science, Indianapolis, IN), according to the manufacturer's instructions. Templates were digested with DNase I and the cRNA purified by LiCl precipitation. Sections were treated with 0.2 M HCl and then acetylated with 0.25% acetic anhydrate in 0.1 M triethanolamine, and prehybridized with hybridization buffer (50% formamide, 2×SSC, 10% dextran sulfate, 0.5 mg/ml sperm DNA, 0.25 mg/ml yeast tRNA, 0.2 mg/ml BSA, 50 ug/ml Heparin, 2.5 mM EDTA and 0.1% Tween-20) at 60°C for 1 hr. Sections were then hybridized with DIG-labeled probes diluted 1∶50 in hybridization buffer (50 µl/section) at 60°C overnight. Sections were washed with 2×SSC at R.T., 1XSSC at 37°C and then treated with RNase A at the same temperature. After RNase A digestion, sections were washed sequentially with 1×SSC at 37°C, 1×SSC: 50% formamide at 52°C, 0.1×SSC at 52°C and then developed with the DIG Nucleic Acid Detection kit (Roche Applied Science, Indianapolis, IN), in conjunction with sheep anti-digoxigenin-alkaline phosphate conjugated antibody (1∶1000) (Roche) and NBT/BCIP chromogen (1∶50) (Roche) according to the manufacturer's instructions. Sections were mounted with mounting medium (VetctaMount™ AQ, Vector Laboratories, Burlingame, CA) and coverslipped with glass.

### Flow cytometry

Intact nuclei were prepared from up to 3 gram of frozen-thawed tissue as described above, with the exception of the fixation step, and further purified by ultracentrifugation through a sucrose cushion at 25,000×*g* for 2.5 hrs at 4°C. The pelleted nuclei were dissolved in 1 mL 1×PBS, centrifuged for 5 min at 14,000×*g* , and the nuclei pellets stored at −80°C until further processed. Nuclei were immunolabeled with anti-NeuN antibody (see above) and sorted using a FACSVantage DiVa system (BD Biosciences), the DNA extracted and processed by Methylight PCR as described above.

## Supporting Information

Figure S1 Scatter diagrams and linear association of PMR with age
(0.61 MB PDF)

Table S1 Gene Function and Disease
(0.14 MB DOC)

Table S2 MethyLight reaction details
(0.08 MB PDF)

Table S3 DNA methylation comparison by diagnosis
(0.07 MB PDF)

Table S4 List of human subjects (postmortem samples)
(0.05 MB PDF)
