# Supplementary material for: DNA Methylation in the Human Cerebral Cortex Is Dynamically Regulated throughout the Life Span and Involves Differentiated Neurons
Source: PLoS One. 2007 Sep 19;2(9):e895. doi: 10.1371/journal.pone.0000895 (PMC1964879; doi:10.1371/journal.pone.0000895)
Supplement: Figure S1 — Scatter diagrams and linear association of PMR with age (0.61 MB PDF) [file pone.0000895.s001.pdf]

FIGURE S1. Scatter diagrams and linear association of PMR value with age. For each gene we summarize the association between PMR value (log-transformed) and age using linear regression models. We fit lines that change slope at a change point. The change point is estimated from the data using a maximum R^2-criterion. If the change in slope is statistically significant at the 0.05-level, we report the result from the two-slope analysis. Otherwise results are reported for a straight line. The non-linear effect for MGMT and sex-specific effect for AR are reported separately. Models are adjusted for diagnosis and sex as indicated. Case 2763 was omitted as an outlier.

Scattergrams are shown on the following pages.

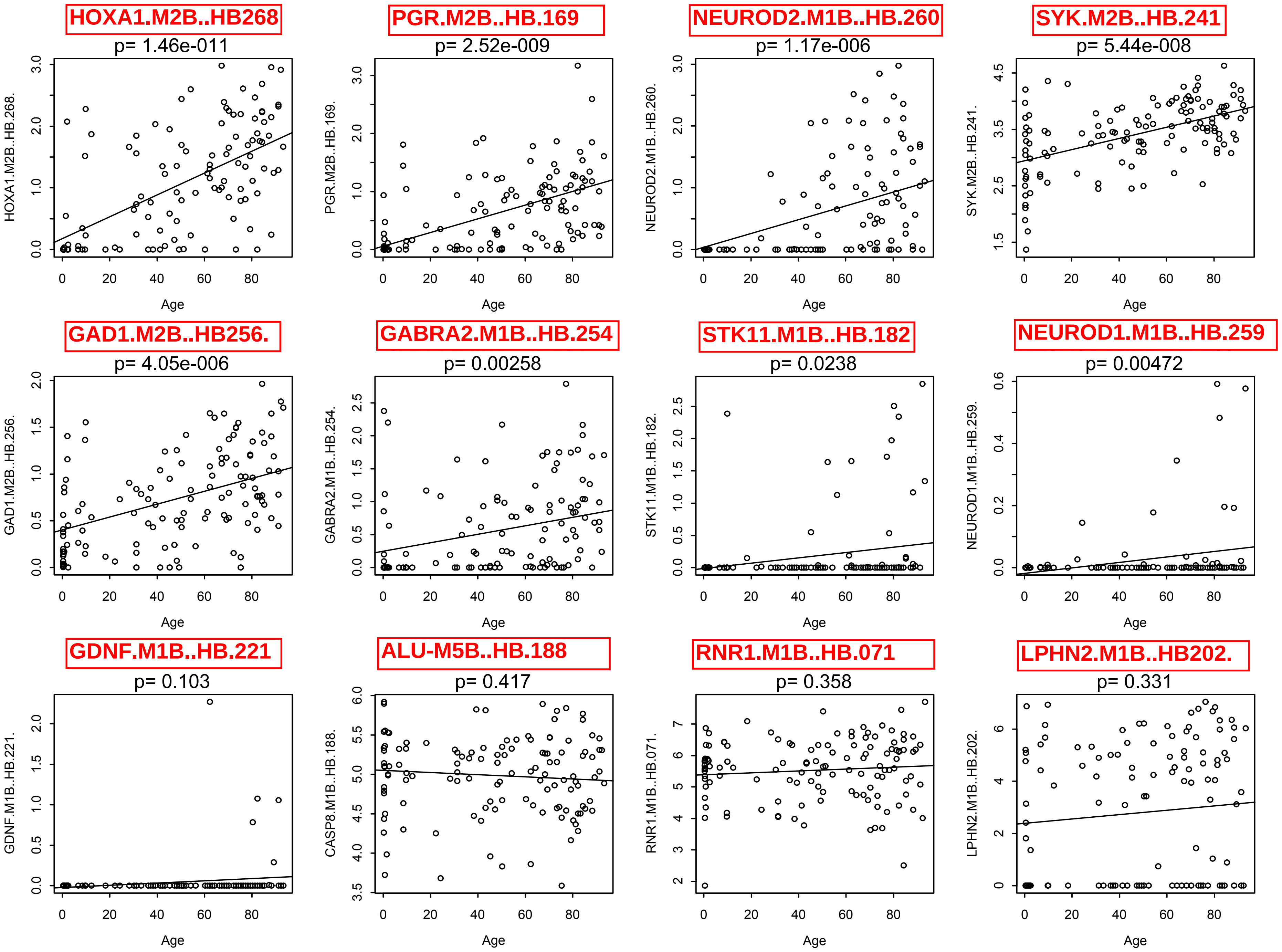

HOXA1.M2B..HB268
p= 1.46e-011
HOXA1.M2B..HB.268.
PGR.M2B..HB.169
p= 2.52e-009
PGR.M2B..HB.169.
NEUROD2.M1B..HB.260
p= 1.17e-006
NEUROD2.M1B..HB.260.
SYK.M2B..HB.241
p= 5.44e-008
SYK.M2B..HB.241.
GAD1.M2B..HB256.
p= 4.05e-006
GAD1.M2B..HB.256.
GABRA2.M1B..HB.254
p= 0.00258
GABRA2.M1B..HB.254.
STK11.M1B..HB.182
p= 0.0238
STK11.M1B..HB.182.
NEUROD1.M1B..HB.259
p= 0.00472
NEUROD1.M1B..HB.259.
GDNF.M1B..HB.221
p= 0.103
GDNF.M1B..HB.221.
ALU-M5B..HB.188
p= 0.417
CASP8.M1B..HB.188.
RNR1.M1B..HB.071
p= 0.358
RNR1.M1B..HB.071.
LPHN2.M1B..HB202.
p= 0.331
LPHN2.M1B..HB.202.
Age

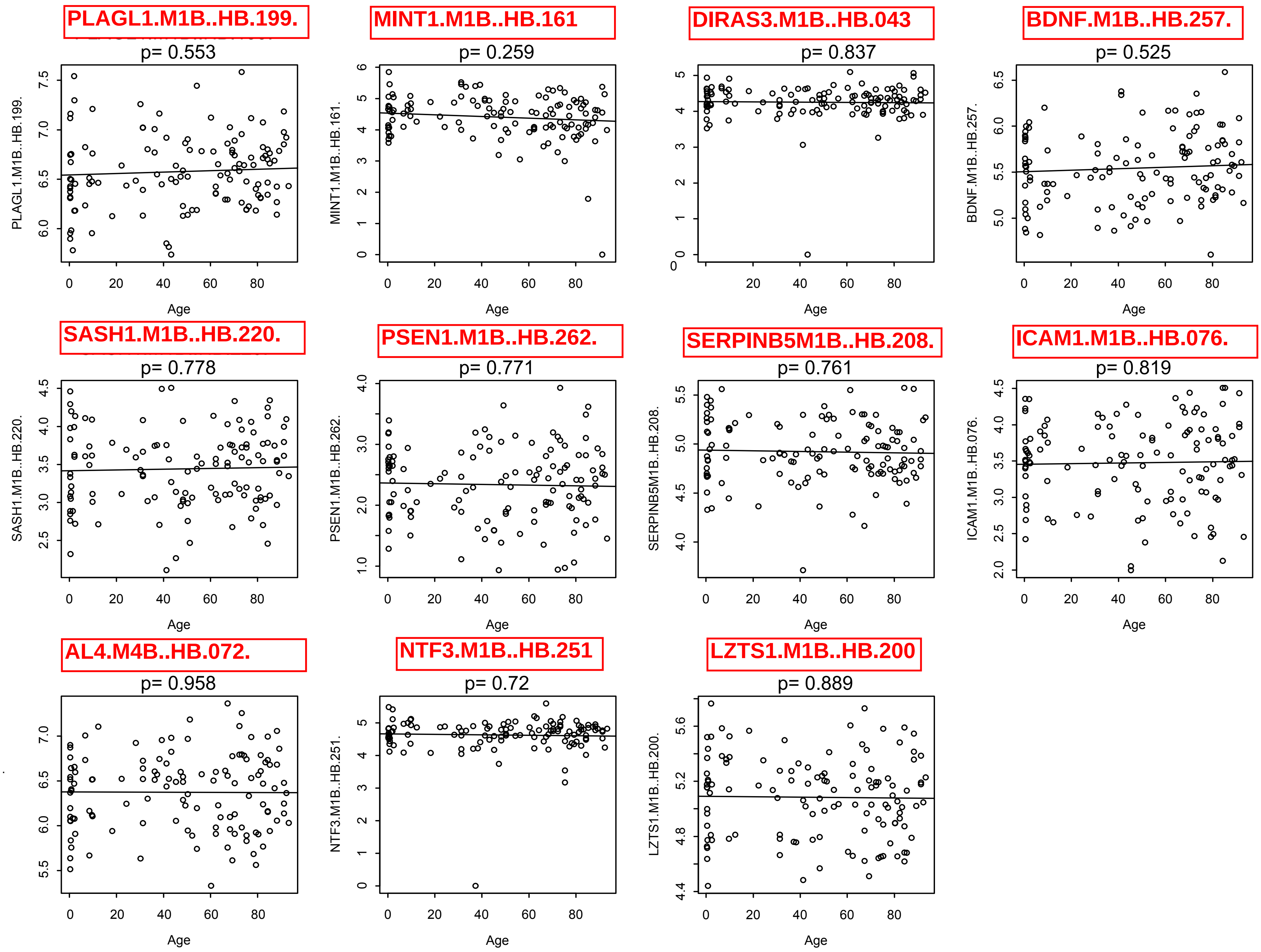

PLAGL1.M1B..HB.199.
p= 0.553
PLAGL1.M1B..HB.199.
Age
MINT1.M1B..HB.161
p= 0.259
MINT1.M1B..HB.161.
Age
DIRAS3.M1B..HB.043
p= 0.837
Age
BDNF.M1B..HB.257.
p= 0.525
BDNF.M1B..HB.257.
Age
SASH1.M1B..HB.220.
p= 0.778
SASH1.M1B..HB.220.
Age
PSEN1.M1B..HB.262.
p= 0.771
PSEN1.M1B..HB.262.
Age
SERPINB5M1B..HB.208.
p= 0.761
SERPINB5M1B..HB.208.
Age
ICAM1.M1B..HB.076.
p= 0.819
ICAM1.M1B..HB.076.
Age
AL4.M4B..HB.072.
p= 0.958
Age
NTF3.M1B..HB.251
p= 0.72
NTF3.M1B..HB.251.
Age
LZTS1.M1B..HB.200
p= 0.889
LZTS1.M1B..HB.200.
Age

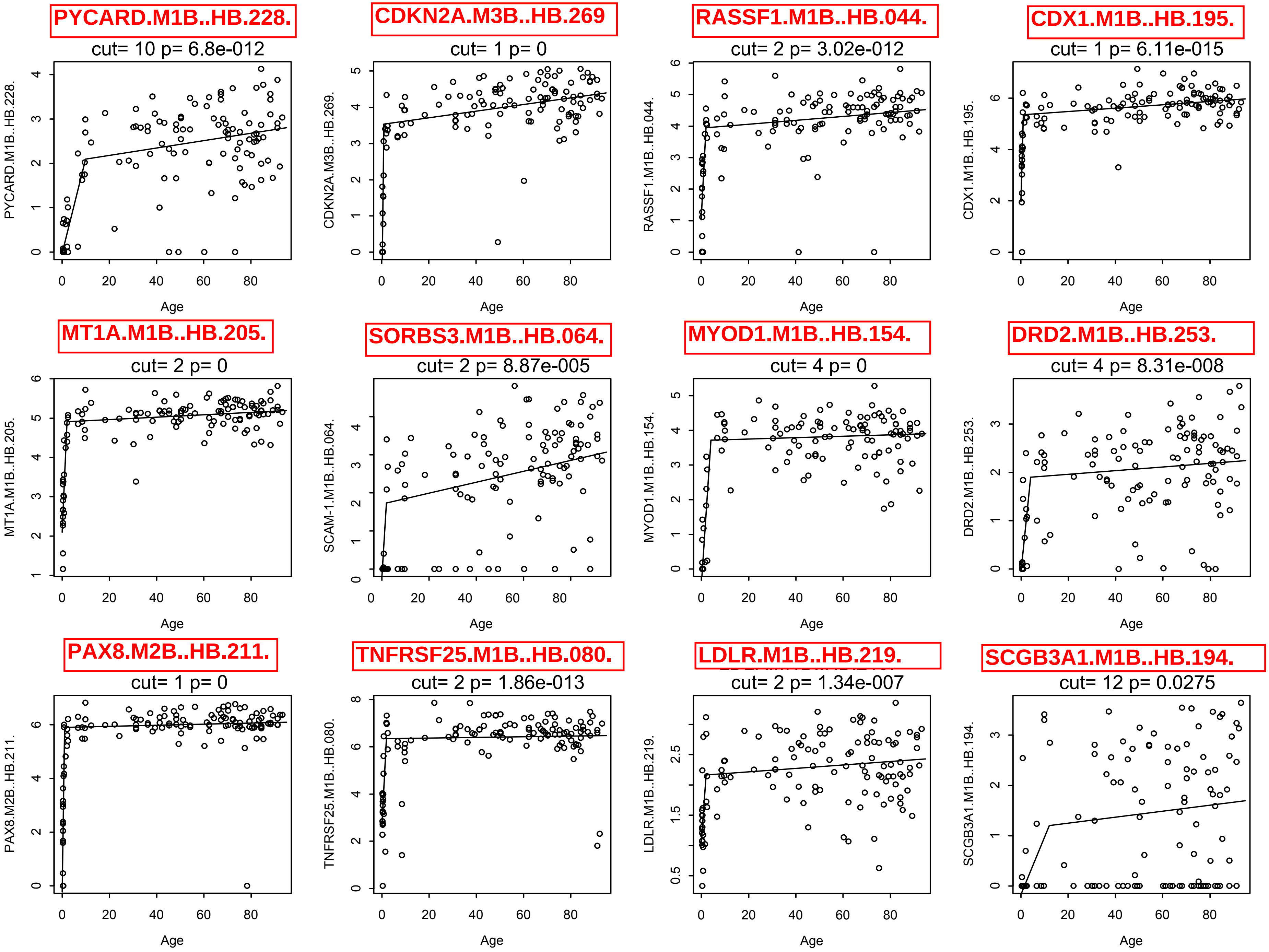
PYCARD.M1B..HB.228.
cut= 10 p= 6.8e-012
PYCARD.M1B..HB.228.
Age
CDKN2A.M3B..HB.269
cut= 1 p= 0
CDKN2A.M3B..HB.269.
Age
RASSF1.M1B..HB.044.
cut= 2 p= 3.02e-012
RASSF1.M1B..HB.044.
Age
CDX1.M1B..HB.195.
cut= 1 p= 6.11e-015
CDX1.M1B..HB.195.
Age
MT1A.M1B..HB.205.
cut= 2 p= 0
MT1A.M1B..HB.205.
Age
SORBS3.M1B..HB.064.
cut= 2 p= 8.87e-005
SCAM-1.M1B..HB.064.
Age
MYOD1.M1B..HB.154.
cut= 4 p= 0
MYOD1.M1B..HB.154.
Age
DRD2.M1B..HB.253.
cut= 4 p= 8.31e-008
DRD2.M1B..HB.253.
Age
PAX8.M2B..HB.211.
cut= 1 p= 0
PAX8.M2B..HB.211.
Age
TNFRSF25.M1B..HB.080.
cut= 2 p= 1.86e-013
TNFRSF25.M1B..HB.080.
Age
LDLR.M1B..HB.219.
cut= 2 p= 1.34e-007
LDLR.M1B..HB.219.
Age
SCGB3A1.M1B..HB.194.
cut= 12 p= 0.0275
SCGB3A1.M1B..HB.194.
Age

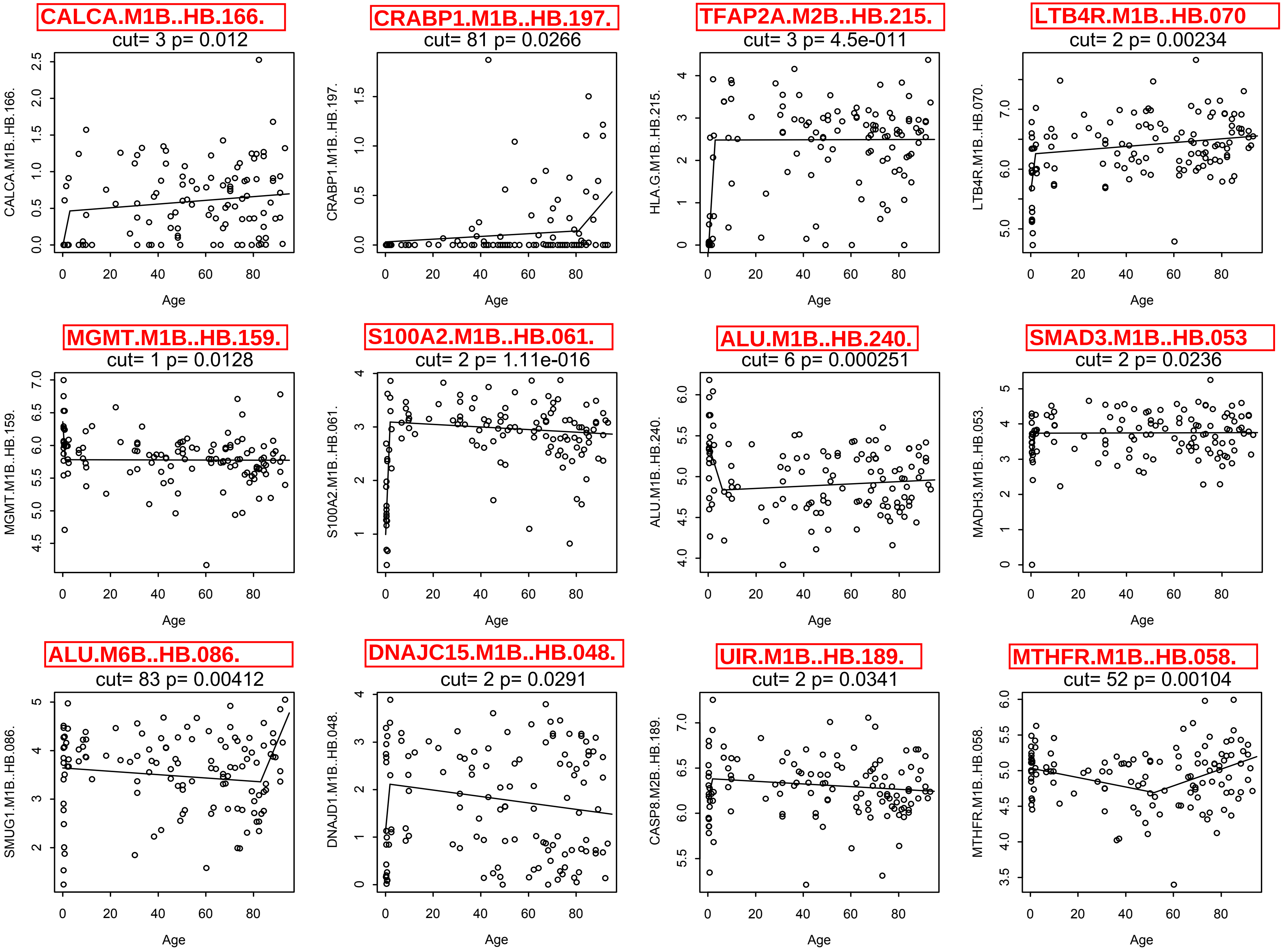
CALCA.M1B..HB.166.
cut= 3 p= 0.012
CALCA.M1B..HB.166.
Age
CRABP1.M1B..HB.197.
cut= 81 p= 0.0266
CRABP1.M1B..HB.197.
Age
TFAP2A.M2B..HB.215.
cut= 3 p= 4.5e-011
HLA.G.M1B..HB.215.
Age
LTB4R.M1B..HB.070
cut= 2 p= 0.00234
LTB4R.M1B..HB.070.
Age
MGMT.M1B..HB.159.
cut= 1 p= 0.0128
MGMT.M1B..HB.159.
Age
S100A2.M1B..HB.061.
cut= 2 p= 1.11e-016
S100A2.M1B..HB.061.
Age
ALU.M1B..HB.240.
cut= 6 p= 0.000251
ALU.M1B..HB.240.
Age
SMAD3.M1B..HB.053
cut= 2 p= 0.0236
MADH3.M1B..HB.053.
Age
ALU.M6B..HB.086.
cut= 83 p= 0.00412
SMUG1.M1B..HB.086.
Age
DNAJC15.M1B..HB.048.
cut= 2 p= 0.0291
DNAJD1.M1B..HB.048.
Age
UIR.M1B..HB.189.
cut= 2 p= 0.0341
CASP8.M2B..HB.189.
Age
MTHFR.M1B..HB.058.
cut= 52 p= 0.00104
MTHFR.M1B..HB.058.
Age

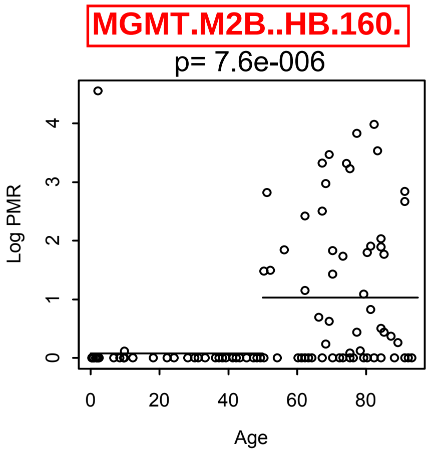

MGMT.M2B..HB.160.
p= 7.6e-006
Log PMR
0
1
2
3
4
0
20
40
60
80
Age

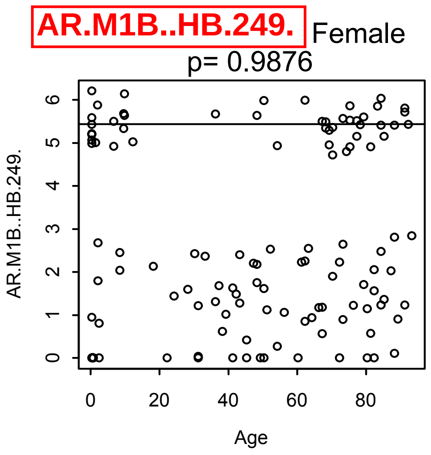

AR.M1B..HB.249.
Female
p= 0.9876
AR.M1B..HB.249.
Age

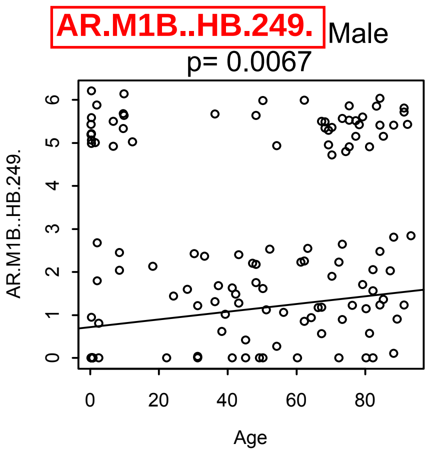

AR.M1B..HB.249.
Male
p= 0.0067
AR.M1B..HB.249.
Age

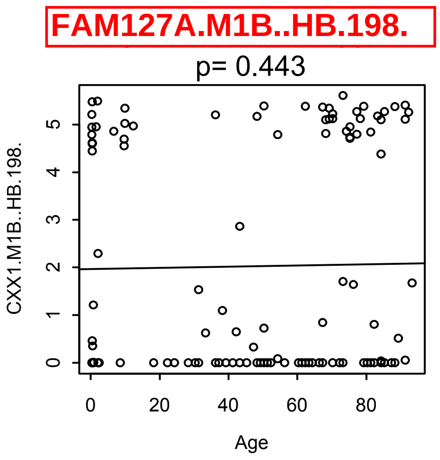

FAM127A.M1B..HB.198.
p= 0.443
CXX1.M1B..HB.198.
Age
