# Supplementary material for: DNA Methylation in the Human Cerebral Cortex Is Dynamically Regulated throughout the Life Span and Involves Differentiated Neurons
Source: PLoS One. 2007 Sep 19;2(9):e895. doi: 10.1371/journal.pone.0000895 (PMC1964879; doi:10.1371/journal.pone.0000895)
Supplement: Table S1 — Gene Function and Disease (0.14 MB DOC) [file pone.0000895.s002.doc]

Table S1

**Gene Function and Disease**

**green = CNS differentiation and neurotransmission; red = Cancer-related**

* (+) transcript expressed in adult cerebral cortex ([www.brainatlas.org/aba/](http://www.brainatlas.org/aba/)) and references in PubMed

(?) expression uncertain or non-detectable in cerebral cortex

| **Gene** | **Gene Number (OMIM)** | **Location** | **Biological Function** | **Expression in Cerebral Cortex*** | **Disease Relevance** |
| --- | --- | --- | --- | --- | --- |
| **ALU** | N/A | N/A | DNA repeat sequence (SINE); 3% total DNA | N/A |  |
| **AR** | 313700 | Xq11.2-q12 | Androgen receptor | + | Increased expression in malignant meningioma; hypermethylated in prostate & endometrial cancer |
| **DIRAS3** | ARHC? 165380; 165370; 165390 | 1p31 | GTPase | + | Hypermethylated in breast, ovarian, & lung cancer |
| **BDNF** | 113505 | 11p13 | Brain derived neurotrophic factor | + | Neuropsychiatric disorders (Mood disorders, schizophrenia and many others) |
| **CALCA** | 114130 | 11p15.2-p15.1 | Calcium-modulating thyroid hormone | + | Hypermethylated in multiple cancers |
| **CASP8** | 601763 | 2q33-q34 | Pro-apoptotic cysteine protease | + | Hypermethylated in neuroblastoma & liver cancer |
| **CDKN2A** | 600160 | 9p21 | Cell cycle regulator | + | Hypermethylated in pituitary tumors, leukemia, & lymphoma |
| **CDX1** | 600746 | 5q31-q33 | Transcription factor for enterocyte differentiation | ? | Hypermethylated in colorectal cancer |
| **CRABP1** | 180230 | 15q24 | Retinoic acid carrier protein | ? | Hypermethylated in thyroid, esophageal, & colorectal cancer |
| **CXX1 (CAAX box 1)** | 300213 | Xq26 | Unknown functions, contains CAAX motif for possible prenylation | Fetal period | Hypermethylated in colorectal cancer |
| **DNAJD1 / DNAJC15 / MCJ** | 29103  (NCBI gene ID) | 13q13 | Type II transmembrane co-chaperone in the Golgi network | + | Hypermethylated in neuroectodermal tumors, ependymomas, ovarian cancer & Wilm’s tumors |
| **DRD2** | 126450 | 11q23 | Dopamine receptor | + | Neuropsychiatric disorders (Schizophrenia, Tourette’s and others) |
|  |  |  |  |  |  |
| **GABRA2** | 137140 | 4p12 | Ligand-gated chloride channel subunit | + | Neuropsychiatric disorders |
| **GAD1** | 605363 | 2q31 | Rate-limiting enzyme in GABA synthesis | + | Neuropsychiatric disorders (Autism, Schizophrenia, Mood and Anxiety Disorders) |
| **GDNF** | 600837 | 5p31.1-p12 | Glial cell derived neurotrophic factor | + | Hirschsprung disease |
| **HLA-G** | 142871 | 6p21.3 | Major histocompatibility complex (MHC) class I molecule | Expressed in cerebral cortex in conjunction with inflammation | Increased expression in glioblastoma, clear cell carcinoma, and melanoma |
| **HOXA1**  **_____ _** | 142955 | 7p15 | Transcription factor | + | Genetic association with autism; increased expression in lung cancer and decreased in breast cancer |
| **ICAM1** | 147840 | 19p13.3-p13.2 | Cell adhesion | + | Decreased expression in ovarian cancer; increased expression in Alzheimer’s disease |
| **LDLR** | 606945 | 19p13.3 | Low density lipoprotein receptor | + | LDLR: Low density lipoprotein receptor-related proteins have been implicated in Alzheimer’s. |
| **LPHN2 / LPHH1** | 607018 | 1p31.1 | G-protein coupled receptor involved in cell adhesion | + | Decreased expression in lung cancer; increased & decreased in breast cancer cell lines |
| **LTB4R** | 601531 | 14q11.2-q12 | Leukotriene B4 receptor | + | Hypermethylated in ovarian cancer ovarian; increased expression in multiple non-CNS cancers |
| **LZTS1** | 606551 | 8p22 | Cell-cycle regulation | + | Hypermethylated in gastric cancer; deleted or mutated in multiple non-CNS cancers |
| **MGMT** | 156569 | 10q26 | DNA repair of O6-alkyl-guanine | + | Hypermethylated in glioblastomas and in multiple non-CNS cancers |
| **MINT1 / APBA1**  **______________** | 602414 | 5q13-14 | Neuronal adaptor protein; synaptic vesicle docking & fusion | + | Hypermethylated in colorectal, gastric, liver, & oral cancer |
| **MT1A** | 156350 | 16q13 | Zinc & copper homeostasis; detoxification of heavy metals; protection against reactive oxygen species | + | Hypermethylated in astrocytoma, breast, & liver cancer |
| **MTHFR** | 607093 | 1p36.3 | Catalyzes the conversion of 5,10-methylenetetrahydrofolate to 5,10-methyltetrahydrofolate | + | Hypermethylated in ovarian cancer; genetic association with colorectal, breast, & ovarian cancer; genetic association with depression and schizophrenia |
| **MYOD1** | 159970 | 11p15.4 | Transcription factor for myogenic differentiation | + | Hypermethylated in astrocytoma; hypermethylated in non-CNS cancer (colorectal, liver, leukemia) |
| **NEUROD1**  **_________** | 601724 | 2q32 | Basic helix-loop-helix (bHLH) transcription factor | + | Genetic association with type II diabetes; expressed in medulloblastoma |
| **NEUROD2** | 601725 | 17q12 | Basic helix-loop-helix (bHLH) transcription factor | + | Expressed in some medulloblastomas |
| **NTF3** | 162660 | 12p13 | Neurotrophin 3 | + | Genetic association with schizophrenia |
| **PAX8** | 167415 | 2q12 | Transcription factor | ? | Genetic association with thyroid cancer |
| **PGR** | 607311 | 11q22-q23 | Progesterone receptor | + | Hypermethylated in breast cancer; decreased expression in endometrial cancer |
| **PLAG1** | 603026 | 6q24-q25 | Zinc-finger transcription factor | + | Overexpressed in pleomorphic adenomas of the salivary gland (translocation); hypermethylated in ovarian cancer |
| **PSEN1** | 104311 | 14q24.3 | Amyloid precursor protein degradation | + | Overexpressed in Alzheimer’s disease |
| **PYCARD** | 606838 | 16p12-p11.2 | Proapoptotic caspase-recruitment domain protein | ? | Hypermethylated in glioblastoma, prostate, breast, & ovarian cancer |
| **RASSF1** | 605082 | 3p21.3 | Cell cycle regulator | ? | Hypermethylated in neuroblastoma, glioma, lung, breast, and prostate cancer |
| **RNR1** | 180450 | 13p12 | Translation of mRNA to protein | ? | Hypermethylated in ovarian cancer |
| **SERPINB5 / MASPIN** | 154790 | 18q21.3 | Serine protease inhibitor | + | Hypermethylated in breast and thyroid cancers; hypomethylated in ovarian cancer |
| **STK11** | 602216 | 19p13.3 | Serine threonine kinase | + | Hypermethylated in Peutz-Jeghers syndrome |
| **SYK** | 600085 | 9q22 | Tyrosine kinase | + | Hypermethylated in gastric and liver cancer |
| **SCAM1 / SORBS3** | 610795 | 8p21 | Cell adhesion | + | Expression levels linked to imatinib response in CML patients |
| **TNFRSF25** | 603366 | 1p36.2 | Cytokine | + | Hypermethylated in bladder cancer |
| **SASH1** | 607955 | 6q24.3 | Signal adaptor protein | + | Decreased expression in breast, liver, and colon cancer |
| **SCGB3A1** | 606500 | 5q35-qter | Cytokine | ? | Hypermethylated in testicular cancer |
| **SMAD3** | 603109 | 18q21.1 | TGF-β effector molecule | + | Decreased expression in T cell ALL |
|  |  |  |  |  |  |
| **S100A2** | 176993 | 1q21 | Calcium-binding protein | + | Hypermethylated in breast and prostate cancer |
