# Supplementary material for: DNA Methylation in the Human Cerebral Cortex Is Dynamically Regulated throughout the Life Span and Involves Differentiated Neurons
Source: PLoS One. 2007 Sep 19;2(9):e895. doi: 10.1371/journal.pone.0000895 (PMC1964879; doi:10.1371/journal.pone.0000895)
Supplement: Table S3 — DNA methylation comparison by diagnosis (0.07 MB PDF) [file pone.0000895.s004.pdf]

Table S3a. Median PMR values by diagnosis in subjects over age 60 years.

RANK	GENE REACTION	Overall (N=57)	Control or Schizophrenia (N=39)	Alzheimer's Disease (N=18)	P-Value	Significance Threshold	
1	SORBS3.M1B..HB.064.	22.55	16.86	38.54	**0.00081**	**0.001**	
2	S100A2.M1B..HB.061.	17.01	20.52	12.92	**0.00197**	**0.002**	
3	LDLR.M1B..HB.219.	9.05	9.77	6.09	0.01	0.003	
4	MYOD1.M1B..HB.154.	52.33	55.19	44.54	0.03	0.004	
5	MGMT.M1B..HB.159.	315.19	318.02	305.60	0.05	0.005	
6	LZTS1.M1B..HB.200.	154.62	169.42	135.27	0.06	0.006	
7	GDNF.M1B..HB.221.	0.00	0.00	0.00	0.07	0.007	
8	PYCARD.M1B..HB.228.	13.33	14.75	8.71	0.07	0.008	
9	STK11.M1B..HB.182.	0.00	0.00	0.00	0.09	0.009	
10	UIR.M1B..HB.189.	498.25	510.27	470.94	0.10	0.01	
11	CRABP1.M1B..HB.197.	0.00	0.00	0.03	0.11	0.011	
12	PLAGL1.M1B..HB.199.	767.14	704.60	818.90	0.15	0.012	
13	DIRAS3.M1B..HB.043.	73.51	69.59	80.06	0.15	0.013	
14	PGR.M2B..HB.169.	1.61	1.73	0.73	0.17	0.014	
15	SERPINB5.M1B..HB.208	127.25	141.58	124.71	0.18	0.015	
16	NEUROD2.M1B..HB.260.	1.45	1.45	2.71	0.19	0.016	
17	GAD1.M2B..HB.256.	1.65	1.40	2.12	0.21	0.017	
18	RNR1.M1B..HB.071.	361.33	422.29	259.07	0.21	0.018	
19	ALU.M1B..HB.086.	34.58	36.39	27.12	0.22	0.019	
20	TFAP2A.M2B..HB.215.	13.24	14.04	11.02	0.26	0.02	
21	MINT1.M1B..HB.161.	68.54	85.67	64.18	0.32	0.021	
22	CDKN2A.M3B..HB.269.	69.16	69.85	67.97	0.33	0.022	
23	NTF3.M1B..HB.251.	115.61	113.10	124.12	0.35	0.023	
24	SASH1.M1B..HB.220.	33.59	32.86	35.35	0.41	0.024	
25	PAX8.M2B..HB.211.	436.52	454.83	402.85	0.41	0.025	
26	SYK.M2B..HB.241.	43.54	44.71	37.65	0.48	0.026	
27	NEUROD1.M1B..HB.259.	0.00	0.00	0.00	0.48	0.027	
28	PSEN1.M1B..HB.262.	10.24	9.21	10.32	0.51	0.028	
29	ALU.M5B..HB.188.	143.89	137.14	148.36	0.52	0.029	
30	GABRA2.M1B..HB.254.	1.00	0.96	1.20	0.52	0.03	*
31	DRD2.M1B..HB.253.	10.15	9.06	10.85	0.53	0.031	
32	LTBR4.M1B..HB.070.	605.31	623.93	603.33	0.56	0.032	*
33	ALU.M1B..HB.240.	143.37	133.04	143.38	0.58	0.033	
34	HOXA1.M2B..HB.268.	3.68	3.68	3.74	0.61	0.034	*
35	CALCA.M1B..HB.166.	0.88	0.73	1.00	0.70	0.035	
36	DNAJC15.M1B..HB.048.	3.54	3.54	2.73	0.71	0.036	
37	SMAD3.M1B..HB.053.	40.45	43.05	38.77	0.71	0.037	
38	CDX1.M1B..HB.195.	344.54	336.10	389.08	0.72	0.038	
39	SCGB3A1.M1B..HB.194.	4.30	3.36	5.43	0.73	0.039	
40	MT1A.M1B..HB.205.	177.26	177.26	178.42	0.76	0.04	
41	TNFRSF25.M1B..HB.080	728.74	728.74	735.68	0.77	0.041	
42	MTHFR.M1B..HB.058.	148.60	147.27	162.26	0.77	0.042	
43	MGMT.M2B..HB.160.	0.45	0.27	0.50	0.82	0.043	
44	FAM127A.M1B..HB.198.	4.18	4.34	0.96	0.85	0.044	**
45	AR.M1B..HB.249.	13.03	16.10	9.92	0.89	0.045	**
46	LPHN2.M1B..HB.202.	57.01	85.38	56.39	0.90	0.046	
47	ALU.M1B..HB.072.	579.37	579.37	564.01	0.95	0.047	
48	RASSF1.M1B..HB.044.	87.72	97.16	86.10	0.96	0.048	
49	BDNF.M1B..HB.257.	274.83	299.93	253.93	0.97	0.049	
50	ICAM1.M1B..HB.076.	32.63	32.63	32.71	0.97	0.05	

*:Adjusted for age.
**:Adjusted for sex.

Table S3b. Median PMR value by diagnosis (Control versus Schizophrenia only) for subjects aged 20 to 90 years.

RANK	GENE REACTION	Overall (N=69)	Control (N=39)	Schizophrenia (N=30)	P-value	Significance Threshold	
1	PAX8.M2B..HB.211.	436.10	402.29	484.92	0.0025	0.001	
2	CDKN2A.M3B..HB.269.	64.87	55.72	76.43	0.02	0.002	
3	MYOD1.M1B..HB.154.	54.89	59.10	49.66	0.04	0.003	
4	BDNF.M1B..HB.257.	249.53	235.61	290.65	0.04	0.004	
5	SERPINB5.M1B..HB.208.	132.63	123.09	140.65	0.06	0.005	
6	RNR1.M1B..HB.071.	287.84	223.74	329.12	0.07	0.006	
7	ALU.M1B..HB.072.	672.23	646.95	687.37	0.07	0.007	
8	LPHN2.M1B..HB.202.	53.71	21.04	94.21	0.09	0.008	
9	SCGB3A1.M1B..HB.194.	2.65	4.77	0.17	0.10	0.009	
10	SORBS3.M1B..HB.064.	12.97	10.86	16.64	0.10	0.01	
11	MT1A.M1B..HB.205.	166.56	162.55	175.92	0.12	0.011	
12	CASP8.M1B..HB.188.	147.61	136.36	180.02	0.14	0.012	
13	LDLR.M1B..HB.219.	10.16	8.54	12.82	0.17	0.013	
14	S100A2.M1B..HB.061.	20.05	20.56	19.20	0.18	0.014	*
15	NEUROD1.M1B..HB.259.	0.00	0.00	0.00	0.21	0.015	
16	NEUROD2.M1B..HB.260.	0.48	0.16	0.54	0.22	0.016	*
17	HOXA1.M2B..HB.268.	2.04	1.74	2.42	0.22	0.017	*
18	DIRAS3.M1B..HB.043.	69.01	63.02	73.72	0.24	0.018	
19	ALU.M1B..HB.240.	129.75	116.22	134.28	0.29	0.019	
20	GABRA2.M1B..HB.254.	0.51	0.28	0.74	0.31	0.02	
21	STK11.M1B..HB.182.	0.00	0.00	0.00	0.33	0.021	
22	ALU.M1B..HB.086.	36.96	36.39	39.96	0.34	0.022	
23	PLAGL1.M1B..HB.199.	690.75	690.75	712.34	0.34	0.023	
24	GDNF.M1B..HB.221.	0.00	0.00	0.00	0.38	0.024	
25	CALCA.M1B..HB.166.	0.76	0.76	0.76	0.39	0.025	
26	AR.M1B..HB.249.	4.52	4.52	4.88	0.41	0.026	***
27	LZTS1.M1B..HB.200.	168.42	162.66	170.82	0.44	0.027	
28	DNAJC15.M1B..HB.048.	5.21	4.07	8.61	0.48	0.028	
29	UIR.M1B..HB.189.	559.41	559.41	572.34	0.48	0.029	
30	MGMT.M2B..HB.160.	0.00	0.00	0.00	0.49	0.03	*
31	PYCARD.M1B..HB.228.	14.67	13.96	14.93	0.54	0.031	
32	ICAM1.M1B..HB.076.	32.63	32.64	30.37	0.55	0.032	
33	MADH3.M1B..HB.053.	44.17	43.53	44.49	0.58	0.033	
34	CRABP1.M1B..HB.197.	0.00	0.00	0.00	0.62	0.034	
35	DRD2.M1B..HB.253.	7.79	8.86	6.59	0.65	0.035	
36	LTB4R.M1B..HB.070.	623.93	614.20	650.82	0.71	0.036	
37	GAD1.M2B..HB.256.	1.08	0.96	1.13	0.73	0.037	*
38	SASH1.M1B..HB.220.	29.17	29.78	27.21	0.74	0.038	
39	MINT1.M1B..HB.161.	90.53	82.41	93.32	0.75	0.039	*
40	RASSF1.M1B..HB.044.	79.48	78.37	86.44	0.77	0.04	*
41	NTF3.M1B..HB.251.	106.54	104.42	109.82	0.78	0.041	
42	SYK.M2B..HB.241.	33.82	33.93	33.31	0.78	0.042	*
43	TFAP2A.M2B..HB.215.	13.44	13.84	13.23	0.86	0.043	
44	PSEN1.M1B..HB.262.	8.92	9.00	8.66	0.91	0.044	
45	MGMT.M1B..HB.159.	333.31	326.38	340.34	0.91	0.045	
46	CDX1.M1B..HB.195.	319.30	312.81	322.79	0.92	0.046	*
47	FAM127A.M1B..HB.198.	0.00	0.00	0.00	0.96	0.047	**
48	TNFRSF25.M1B..HB.080.	789.11	732.11	807.76	0.96	0.048	
49	PGR.M2B..HB.169.	1.18	1.31	1.14	0.98	0.049	*
50	MTHFR.M1B..HB.058.	135.10	135.95	134.31	0.99	0.05	

*:Adjusted for age.
**:Adjusted for sex.
***:Adjusted for age and sex.

Table S3 DNA Methylation Comparison by Diagnosis.

We used linear regression and ranked PMR value as the outcome to test for an association between PMR and diagnosis on subsets of subjects, restricted by the age range of the diseased subgroup (Table 4a subjects over 60 years old, Table 4b subjects aged 20 to 90 years).
We adjust for sex for the two X-linked reactions and adjust for age for reactions that show a linear association between age and methylation level in the subset.
