# Supplementary material for: DNA Methylation in the Human Cerebral Cortex Is Dynamically Regulated throughout the Life Span and Involves Differentiated Neurons
Source: PLoS One. 2007 Sep 19;2(9):e895. doi: 10.1371/journal.pone.0000895 (PMC1964879; doi:10.1371/journal.pone.0000895)
Supplement: Table S4 — List of human subjects (postmortem samples) (0.05 MB PDF) [file pone.0000895.s005.pdf]

ID Number	Diagnosis Group	Brain Region (TCX=Temporal Cortex; FCX=Frontal Cortex)	Gender (M=Male; F=Female)	Age (in Years from Conception)
5169	Control	TCX	F	0.3
5170	Control	TCX	F	0.3
5171	Control	TCX	M	0.3
5172	Control	TCX	F	0.3
5173	Control	TCX	M	0.4
5176	Control	TCX	F	0.4
5177	Control	TCX	F	0.4
5180	Control	TCX	F	0.4
5179	Control	TCX	M	0.4
5175	Control	TCX	F	0.4
5178	Control	TCX	M	0.4
5181	Control	TCX	M	0.5
5182	Control	TCX	M	0.6
5183	Control	TCX	M	0.7
5184	Control	TCX	M	0.8
5185	Control	TCX	M	0.8
5186	Control	TCX	M	0.8
5187	Control	TCX	F	1.5
5189	Control	TCX	F	2.0
5190	Control	TCX	M	2.1
5191	Control	TCX	M	2.1
5192	Control	TCX	M	2.5
5193	Control	TCX	M	2.5
5194	Control	TCX	F	6.7
5195	Control	TCX	F	6.7
5196	Control	TCX	M	8.6
5197	Control	TCX	M	8.6
5198	Control	TCX	F	9.7
5199	Control	TCX	F	9.7
5200	Control	TCX	F	9.9
5201	Control	TCX	F	9.9
5202	Control	TCX	F	12.3
5203	Control	TCX	M	18.3
5162	Control	TCX	M	22.3
5159	Schizophrenia	TCX	M	24.3
5167	Schizophrenia	TCX	M	28.3
5136	Control	TCX	M	30.3
5098	Control	FCX	M	31.3
5127	Control	TCX	M	31.3
5145	Control	TCX	M	31.3
5204	Control	TCX	M	31.3
5153	Schizophrenia	TCX	M	33.3
5095	Control	FCX	F	36.3
5125	Schizophrenia	TCX	M	36.3
5205	Control	TCX	M	37.3
5135	Control	TCX	M	38.3
5121	Control	TCX	M	39.3
5137	Control	TCX	M	41.3
5165	Schizophrenia	TCX	M	41.3
5166	Control	TCX	M	42.3
5126	Schizophrenia	TCX	M	43.3
5129	Schizophrenia	TCX	M	43.3
5104	Schizophrenia	TCX	M	45.3
5124	Schizophrenia	TCX	M	45.3
5110	Schizophrenia	TCX	M	47.3
5114	Control	TCX	M	48.3
5148	Schizophrenia	TCX	F	48.3
5130	Schizophrenia	TCX	M	48.3
5112	Control	TCX	M	49.3
5099	Control	FCX	M	50.3
5106	Schizophrenia	TCX	F	50.3
5134	Schizophrenia	TCX	M	50.3
5163	Schizophrenia	TCX	M	51.3
2773	Control	TCX	M	52.3
5100	Control	FCX	F	54.3
5138	Control	TCX	M	54.3
5158	Schizophrenia	TCX	M	56.3
2768	Alzheimer's	TCX	M	60.3
5164	Schizophrenia	TCX	M	61.3
5154	Control	TCX	M	62.3
2766	Alzheimer's	TCX	F	62.3
2777	Alzheimer's	TCX	M	62.3
2790	Control	TCX	M	63.3
5140	Control	TCX	M	64.3
5120	Control	TCX	M	66.3
5122	Control	TCX	M	67.3
5103	Schizophrenia	TCX	F	67.3
5108	Schizophrenia	TCX	M	67.3
5156	Control	TCX	F	68.3
5152	Schizophrenia	TCX	F	68.3
5115	Control	TCX	F	69.3
5132	Control	TCX	F	69.3
5150	Control	TCX	F	70.3
5157	Schizophrenia	TCX	F	70.3
5109	Schizophrenia	TCX	M	70.3
2767	Control	TCX	M	72.3
5142	Schizophrenia	TCX	M	72.3
2780	Control	TCX	F	73.3
5139	Schizophrenia	TCX	M	73.3
2783	Alzheimer's	TCX	M	73.3
2765	Alzheimer's	TCX	F	74.3
5111	Control	TCX	F	75.3
2779	Control	TCX	F	75.3
5107	Schizophrenia	TCX	F	75.3
5141	Control	TCX	M	76.3
2764	Alzheimer's	TCX	F	77.3
2786	Alzheimer's	TCX	F	77.3
2776	Alzheimer's	TCX	F	78.3
5117	Control	TCX	F	79.3
5151	Control	TCX	M	79.3
5161	Schizophrenia	TCX	M	80.3
2787	Alzheimer's	TCX	M	80.3
2775	Control	TCX	F	81.3
5144	Control	TCX	M	81.3
5143	Control	TCX	M	82.3
5155	Schizophrenia	TCX	M	82.3
2762	Alzheimer's	TCX	M	82.3
2781	Alzheimer's	TCX	M	82.3
5102	Schizophrenia	TCX	F	83.3
5118	Schizophrenia	TCX	F	84.3
5168	Schizophrenia	TCX	M	84.3
2774	Alzheimer's	TCX	F	84.3
2761	Alzheimer's	TCX	M	84.3
5116	Control	TCX	F	85.3
2785	Alzheimer's	TCX	M	85.3
2772	Alzheimer's	TCX	M	87.3
5123	Control	TCX	F	88.3
2769	Alzheimer's	TCX	M	88.3
2784	Alzheimer's	TCX	M	89.3
5149	Control	TCX	M	91.3
2770	Control	TCX	F	91.3
2788	Alzheimer's	TCX	F	91.3
5096	Control	FCX	F	92.3
5097	Control	FCX	M	93.3
2763	Control	TCX	M	104.3

Table S4: List of Human Subjects
