# Supplementary material for: DNA Methylation in the Human Cerebral Cortex Is Dynamically Regulated throughout the Life Span and Involves Differentiated Neurons
Source: PLoS One. 2007 Sep 19;2(9):e895. doi: 10.1371/journal.pone.0000895 (PMC1964879; doi:10.1371/journal.pone.0000895)
Supplement: Table S2 — MethyLight reaction details (0.08 MB PDF) [file pone.0000895.s003.pdf]

HUGO GENE NAME (IF AVAILABLE)	REACTION ID	REACTION DESIGN CODE	ALIASES	CHROMOSOMAL LOCATION	GENBANK ACCESSION NUMBER	UNIGENE NUMBER	AMPLICON LOCATION (GENBANK NUMBERING)	FORWARD PRIMER SEQUENCE	REVERSE PRIMER SEQUENCE	PROBE OLIGO SEQUENCE	SOURCE
**DIRAS3**	DIRAS3-M1B	HB-043	DIRAS family, GTP-binding RAS-like 3; ARHI; NOEY2	1p31	AF202543	Hs.194695	1953-2038	GCGTAAGCGGAATTTATGTTTGT	CCGCGATTTTATATTCCGACTT	6FAM-CGCACAAAAACGAAATACGAAAACGCAAA-BHQ-1	Previously described as *ARHI* in Fiegl, H. et al Cancer Epidemiol BioMark Prev **13**,882-888 (2004)
**RASSF1**	RASSF1-M1B	HB-044	Ras association (RalGDS/AF-6) domain family 1	3p21.3	AC002481	Hs.476270	18107-18171	ATTGAGTTGCGGGAGTTGGT	ACACGCTCCAACCGAATACG	6FAM-CCCTTCCCAACGCGCCCA-BHQ-1	Previously described as *RASSF1A* in Ehrlich, M. et al. *Oncogene* **21**, 6694-6702 (2002).
**DNAJC15**	DNAJC15-M1B	HB-048	DnaJ (Hsp40) homolog, subfamily C, member 15; MCJ; DNAJD1	13q13	AL445217	Hs.438830	68466-68546	TTTCGGGTCGTTTTGTTATGG	ACTACAAATACTCAACGTAACGCAAACT	6FAM-TCGCCAACTAAAACGATAACACCACGAACA-BHQ-1	Previously described as *MCJ* in Ehrlich, M. et al. *Oncogene* **21**, 6694-6702 (2002).
**SMAD3**	SMAD3-M1B	HB-053	SMAD family member 3; MADH3/Mothers against DPP homolog 3 (Drosophila)	15q22-q23	AC087482	Hs.36915	160470-160544	CGTGAAGCGTTTGTTGGGT	TTAACCGCCTTCTCGCACC	6FAM-TCCTCCTACCCGTTCTACTCGCCCTTCTT-BHQ-1	Previously described as *MADH3* in Ehrlich, M. et al. *Oncogene* **21**, 6694-6702 (2002).
**MTHFR**	MTHFR-M1B	HB-058	5,10-Methylenetetrahydrofolate Reductase (NADPH)	1p36.3	AF398930	Hs.214142	10625-10715	TGGTAGTGAGAGTTTTAAAGATAGTTCG	CGCCTCATCTTCTCCCGA	6FAM-TCTCATACCGCTCAAAATCCAAACCCG-BHQ-1	Eads, C.A. et al. *Cancer Res* **61**, 3410-3418 (2001).
**S100A2**	S100A2-M1B	HB-061	S100 Calcium-binding Protein A2; S100L	1q21	Y07755	Hs.516484	1388-1469	TGTTTGAGTCGTAAGTAGGGCGT	CGTATCATTACAATACCGACCTCCT	6FAM-ATCCTCCCTTTCTTATCCGCCAAACCCT-BHQ-1	Muller, H.M. et al. *Cancer Lett***209**, 231-236 (2004).
**SORBS3**	SORBS3-M1B	HB-064	Sorbin and SH3 domain containing 3; SCAM-1	8p21	AC037459	Hs.528572	86568-86640	GTTTCGGTTGTCGTTGGGTT	ACGCCGACGAACTCTACGC	6FAM-ACGACGCAATCAAAACCCGCGA-BHQ-1	Previouly described as SCAM-1 in Weisenberger, D.J. et al Nature Genet 38, 787-793 (2006).
**LTB4R**	LTB4R-M1B	HB-070	Leukotriene B4 Receptor; BLT1	14q11.2-q12	AB008193	Hs.655431	2257-2332	GCGTTGGTTTTATCGGAAGG	AAACCGTAATTCCCGCTCG	6FAM-GACTCCGCCCAACTTCGCCAAAA-BHQ-1	Previously described as *BLT1* in Muller, H.M. et al *Ann NY Acad Sci* **1022**, 44-49 (2004).
**RNR1**	RNR1-M1B	HB-071	Ribosomal RNA	13p12	X01547	N/A	219-293	CGTTTTGGAGATACGGGTCG	AAACAACGCCGAACCGAA	6FAM-ACCGCCCGTACCACACGCAAA-BHQ-1	Widschwendter, M. et al. *Cancer Res***64**, 3807-3813 (2004).
**ALU**	ALU-M4B	HB-072	Alu Repeat (Designed against Alu repeat at 9q34.1)	N/A	AL441992	N/A	118456-118558	AGGTCGAGGTCGGCGG	CCACGCCCGACTAATTTTATATCTT	6FAM-CAAACTAATCTCAAACTCCCGACCTCAAACGA-BHQ-1	GenBank Number: AL441992; Amplicon Location: 118456-118558.
**ICAM1**	ICAM1-M1B	HB-076	Intercellular Adhesion Molecule 1	19p13.3-p13.2	AC011511	Hs.643447	85597-85676	GGTTAGCGAGGGAGGATGATT	TCCCCTCCGAAACAAATACTACAA	6FAM-TTCCGAACTAACAAAATACCCGAACCGAAA-BHQ-1	Ehrlich, M. et al. *Oncogene* **21**, 6694-6702 (2002).
**TNFRSF25**	TNFRSF25-M1B	HB-080	Death Receptor 3/TNF Receptor superfamily, member 25/TNFRSF12	1p36.2	AY254324	Hs.462529	1930-1998	GCGGAATTACGACGGGTAGA	ACTCCATAACCCTCCGACGA	6FAM-CGCCCAAAAACTTCCCGACTCCGTA-BHQ-1	Formerly described as *TNFRSF12* in Ehrlich, M. et al. *Oncogene* **21**, 6694-6702 (2002).
**ALU**	ALU-M6B	HB-086	Alu Repeat (Designed against Alu repeat at 12q13.11-q13.3)	N/A	AC023794	N/A	156022-156167	GGATTATAGGCGCGCGTTATT	TCACACCCGTAATCCGAACA	6FAM-ACCGAAACGAACGAATCACGAAATCAAA-BHQ-1	Weisenberger, D.J. et al Nature Genet **38**, 787-793 (2006).
**MYOD1**	MYOD1-M1B	HB-154	Myogenic Determining factor 3; MYF3; PUM	11p15.4	AF027148	Hs.181768	9889-9962	GAGCGCGCGTAGTTAGCG	TCCGACACGCCCTTTCC	6FAM-CTCCAACACCCGACTACTATATCCGCGAAA-BHQ-1	Eads, C.A. et al. *Cancer Res* **61**, 3410-3418 (2001).
**MGMT**	MGMT-M1B	HB-159	O-6 Methylguanine Methyltransferase	10q26	AL355531	Hs.501522	47207-47297	CTAACGTATAACGAAAATCGTAACAACC	AGTATGAAGGGTAGGAAGAATTCGG	6FAM-CCTTACCTCTAAATACCAACCCCAAACCCG-BHQ-1	Eads, C.A. et al. *Cancer Res* **61**, 3410-3418 (2001).
**MGMT**	MGMT-M2B	HB-160	O-6 Methylguanine Methyltransferase	10q26	AL355531	Hs.501522	46928-47010	GCGTTTCGACGTTCGTAGGT	CACTCTTCCGAAAACGAAACG	6FAM-CGCAAACGATACGCACCGCGA-BHQ-1	Virmani, A.K. et al. *Cancer Epidemiol Biomarkers Prev***11**, 291-297 (2002).
**MINT1/SV2C**	MINT1-M1B	HB-161	Methylated in Tumor-1 (located within synaptic vesicle glycoprotein 2C (SV2C)	5q13-14	AC026774	N/A	44288-44415	GGGTTGAGGTTTTTTGTTAGCG	CCCCTCTAAACTTCACAACCTCG	6FAM-CTACTTCGCCTAACCTAACGCACAACAAACG-BHQ-1	Weisenberger, D.J. et al Nature Genet **38**, 787-793 (2006).
**CALCA**	CALCA-M1B	HB-166	Calcitonin	11p15.2-p15.1	X15943	Hs.37058	1707-1807	GTTTTGGAAGTATGAGGGTGACG	TTCCCGCCGCTATAAATCG	6FAM-ATTCCGCCAATACACAACAACCAATAAACG-BHQ-1	Eads, C.A. et al. *Cancer Res* **61**, 3410-3418 (2001).
**PGR**	PGR-M2B	HB-169	Progesterone Receptor B	11q22-q23	AP001533	Hs.368072	111605-111698	TTATAATTCGAGGCGGTTAGTGTTT	TCGAACTTCTACTAACTCCGTACTACGA	6FAM-ATCATCTCCGAAAATCTCAAATCCCAATAATACG-BHQ-1	Virmani, A.K. et al. *Cancer Epidemiol Biomarkers Prev***11**, 291-297 (2002).
**STK11**	STK11-M1B	HB-182	LKB/Serine-Threonine Kinase 11 (Peutz-Jeghers Syndrome)	19p13.3	AC011544	Hs.515005	27020-27112	TGAGGTTCGGGTTTTATTGGAA	GAATCCAAACCCGATACAAAATCT	6FAM-CGAAAATCCGAAACGACGACTCAAACG-BHQ-1	GenBank Number: AC011544; Amplicon Location: 27020-27112.
**ALU**	ALU-M5B	HB-188	Alu Repeat (Designed against Alu repeat at 2q33-q34)	N/A	AC007256	N/A	156130-156221	GGTATGATGGCGTATGTTTGTAATTT	CGACTCACCACAACTTCCACC	6FAM-AAACGATTCTCCTACCTCAACCTCCCGAA-BHQ-1	GenBank Number: AC007256; Amplicon Location: 156130-156221.
**UIR**	UIR-M1B	HB-189	Unidentified interspersed repeat (Designed at 2q33-q34)	N/A	AC007256	N/A	133136-133243	TTAGACGATGGGTGGTTAAGTAGAGAC	CCAACCGCCTACCTTAACCTC	6FAM-CTACCCGACCGCCACGCCG-BHQ-1	GenBank Number: AC007256; Amplicon Location: 133136-133243.
**SCGB3A1**	SCGB3A1-M1B	HB-194	Secretoglobin family 3A member 1; HIN-1; HIN1; LU105; UGRP2	5q35-qter	AC122714	Hs.62492	80825-80911	GGCGTAGCGGGCGTC	CTACGTAACCCTATCCTACAACTCCG	6FAM-CGAACTCCTAACGCGCACGATAAAACCTAA-BHQ	Weisenberger, D.J. et al Nature Genet **38**, 787-793 (2006).
**CDX1**	CDX1-M1B	HB-195	Caudal-Type Homeo Box Transcription Factor 1	5q31-q33	AC005895	Hs.1545	35199-35266	TGAGCGGTTGTTCGTCGTC	AAATCCCCCGCGCATACTA	6FAM-CCTAAAACCGCCGCTACCGACCG-BHQ	Weisenberger, D.J. et al Nature Genet **38**, 787-793 (2006).
**CRABP1**	CRABP1-M1B	HB-197	Cellular Retinoic Acid Binding Protein 1; RBP5; CRABP; CRABPI; CRABP-I	15q24	AC011270	Hs.346950	122142-122223	TCGAAATTTTCGTTGTTGCGT	TATCCGTACCTACCGCCGC	6FAM-ACCATACCCAACTTCGCCGACACCTAA-BHQ	GenBank Number: AC011270; Amplicon Location: 122142-122223.
**FAM127A**	FAM127A-M1B	HB-198	Family with sequence similarity 127, member A , CAAX box 1, CXX1	Xq26	AL136169	Hs.250708	30728-30809	GATGGACGGTCGGGTGTAGTT	AATCGAATTCCTCCAACGACG	6FAM-TCGCAAACCGAATCGACAAAACCAA-BHQ	GenBank Number: AL136169; Amplicon Location: 30728-30809.
**PLAGL1**	PLAGL1-M1B	HB-199	Pleiomorphic Adenoma Gene-Like 1/LOT1/Zac1	6q24-q25	AL109755	Hs.444975	52969-53045	ATCGACGGGTTGAATGATAAATG	CTCGACGCAACCATCCTCTT	6FAM-ACTACCGCGAACGACAAAACCCACG-BHQ-1	Weisenberger, D.J. et al Nature Genet **38**, 787-793 (2006).
**LZTS1**	LZTS1-M1B	HB-200	Leucine Zipper, Putative Tumor Suppressor 1/F37/FEZ1	8p22	AC025853	Hs.521432	24463-24547	GCGGCGTTGTAGGGACG	CGCGCGCTAACTCTTCTACG	6FAM-ATTACCGCCTTTAAACTCCGAACCCTCCA-BHQ-1	Weisenberger, D.J. et al Nature Genet **38**, 787-793 (2006).
**LPHN2**	LPHN2-M1B	HB-202	Latrophilin 1; LEC1; LPHH1; KIAA0786	1p31.1	AL358939	Hs.24212	143237-143336	GAGGATTTAGCGCGTAGTGAGTG	AATCCCCGAACTCTACCTCCA	6FAM-CCCATTAACACACCCATTCAACCGCTAA-BHQ-1	Weisenberger, D.J. et al Nature Genet **38**, 787-793 (2006).
**MT1A**	MT1A-M1B	HB-205	Metallothionein 1A; Metallothionein-I	16q13	AC106779	Hs.655199	18175-18254	CGTGTTTTCGTGTTATTGTGTACG	CTCGCTATCGCCTTACCTATCC	6FAM-TCCACACCTAAATCCCTCGAACCCACT-BHQ-1	Weisenberger, D.J. et al Nature Genet **38**, 787-793 (2006).
**SERPINB5**	SERPINB5-M1B	HB-208	Serine Proteinase Inhibitor, clade B, member 5; PI5; maspin	18q21.3	AC036176	Hs.55279	51709-51788	GAAAAGGAATAGGTAAGCGAGGAGT	ATAAACCACCGCTACTTCTACCCA	6FAM-CACGATCGCCTCCACATCCAAATCTTT-BHQ-1	Weisenberger, D.J. et al Nature Genet **38**, 787-793 (2006).
**PAX8**	PAX8-M2B	HB-211	Paired Box Gene 8	2q12	AC016683	Hs.469728	115663-115758	GTTCGTAGTTCGTCGAGGGTTC	CGCATCTCATACCCTTCTCCTAAAT	6FAM-CAAACGCGACCCGAACCTACGAAAA-BHQ-1	Weisenberger, D.J. et al Nature Genet **38**, 787-793 (2006).
**TFAP2A**	TFAP2A-M2B	HB-215	Transcription factor AP-2 alpha (activating enhancer binding protein 2 alpha)	6p21.3	AL138885	Hs.519880	76163-76288	CACCCCCATATACGCGCTAA	GGTCGTTACGTTTCGGGTAGTTTA	6FAM-CGCGCTCACACGCTCAAAAACCT-BHQ-1	Previously described as *HLA-G* in Muller, H.M. et al *Ann NY Acad Sci* **1022**, 44-49 (2004).
**LDLR**	LDLR-M1B	HB-219	Low Density Lipoprotein Receptor	19p13.3	AC011485	Hs.213289	90572-90674	GATATCGGTTTTTTAATTCGTGAAGTT	TTCACCGAAAACCCAAATACAA	6FAM-ATCAAATCGCCTACCCTAACGACACTTTCG-BHQ-1	Weisenberger, D.J. et al Nature Genet **38**, 787-793 (2006).
**SASH1**	SASH1-M1B	HB-220	SAM and SH3 domain containing 1; KIAA0790	6q23	AL513164	Hs.193133	97419-97530	TGGAAGAGTTTATTTTGAAGAGAGGG	GCGACTCGTTCCTTCTAACAAATC	6FAM-AAACCCGACAAAAATAACCGCGAAACCT-BHQ-1	Weisenberger, D.J. et al Nature Genet **38**, 787-793 (2006).
**GDNF**	GDNF-M1B	HB-221	Glial Cell Derived Neurotrophic Factor	5p13.1-p12	AC008869	Hs.248114	108758-108866	CGGTAGTTGTCGTTGAGTCGTTC	AACAACCGCCGCTACTTTAAATA	6FAM-CGCGCGTCGCGCTCTTAACTAAAA-BHQ-1	Weisenberger, D.J. et al Nature Genet **38**, 787-793 (2006).
**PYCARD**	PYCARD-M1B	HB-228	PYD and CARD domain containing; TMS1; CARD5; ASC1; MGC10332	16p12-p11.2	AC009088	Hs.499094	85425-85330	TTGGAGATTTACGGCGTCG	ACCCTAATACGTAACCGCCTACAA	6FAM-CATCTCCTACAAACCCATATCGCGCAA-BHQ-1	Weisenberger, D.J. et al Nature Genet **38**, 787-793 (2006).
**ALU**	ALU-M1B	HB-240	Alu Repeat (Designed against Alu repeat at 1q21)	N/A	Y07755	N/A	5059-5164	ATTATGTTAGTTAGGATGGTTTCGATTTT	CAATCGACCGAACGCGA	6FAM-CCCAACACTTTAAAAAACCGAAATAAATAAATCACGA-	Weisenberger, D.J. et al Nature Genet **38**, 787-793 (2006).
**SYK**	SYK-M2B	HB-241	Spleen Tyrosine Kinase; LKB1	9q22	AL354862	Hs.371720	50529-50613	AGGGTCGTTGGGTGTTTGTG	AACATAAACCGCATCGATCCC	6FAM-CGCCAACGCGATAACTTCTATAACTACCCAA-BHQ-1	Weisenberger, D.J. et al Nature Genet **38**, 787-793 (2006).
**AR**	AR-M1B	HB-249	Androgen Receptor	Xq11.2-q12	AL049564	Hs.496240	102963-103042	GCGTTTTTTTCGAGATTTCGG	GCCTCCTCTACCTATAAACTTACTCCG	6FAM-ACCGTCCCGCTCTCCCAACAAACTA-BHQ-1	Previously described as *AR1* in Virmani, A.K. et al. *Cancer Epidemiol Biomarkers Prev***11**, 291-297 (2002).
**NTF3**	NTF3-M1B	HB-251	Neurotrophin 3	12p13	AC135585	Hs.99171	7503-7576	TTTCGTTTTTGTATTTTATGGAGGATT	CCGTTTCCGCCGTAATATTC	6FAM-TCGCCACCACGAAACTACCCACG-BHQ-1	Weisenberger, D.J. et al Nature Genet **38**, 787-793 (2006).
**DRD2**	DRD2-M1B	HB-253	Dopamine receptor D2	11q23	AP002840	Hs.73893	110939-111008	GAAGTCGGAAATTTTGGTCGC	ATCTCGAAAAAACACTTCCCCC	6FAM-ACACCCAAACGCGAAACCCGAAACT-BHQ-1	Weisenberger, D.J. et al Nature Genet **38**, 787-793 (2006).
**GABRA2**	GABRA2-M1B	HB-254	Gamma-Aminobutyric Acid A Receptor, Alpha 2	4p12	AC095060	Hs.116250	42382-42455	TCGTCGGAGGAGCGGA	AACCTCTCGAAAACCCCAACA	6FAM-ACGACCTCGAAAAACAACCCGAAACTACG-BHQ-1	Weisenberger, D.J. et al Nature Genet **38**, 787-793 (2006).
**GAD1**	GAD1-M2B	HB-256	Glutamine Decarboxylase 1	2q31	AC007405	Hs.420036	70850-70932	CGATTGGTTCGGCGTAGAAA	CCCTCCGATATACAAAACCCC	6FAM-CCCGCACAACTCTCGCTTCTCTTTACAA-BHQ-1	Weisenberger, D.J. et al Nature Genet **38**, 787-793 (2006).
**BDNF**	BDNF-M1B	HB-257	Brain Derived Neurotrpohic Factor	11p13	AC103796	Hs.502182	26222-26291	AGTGGTTGCGTAAGTTGGGAA	TCATCCTTTCGCCTCCAAAT	6FAM-CTTTCCGCCAACACGTAACCTCTTCG-BHQ-1	GenBank Number: AC103796; Amplicon Location: 26222-26291.
**NEUROD1**	NEUROD1-M1B	HB-259	Neurogenic Differentiation 1	2q32	AC013733	Hs.693737	78576-78657	GTTTTTTGCGTGGGCGAAT	CCGCGCTTAACATCACTAACTAAA	6FAM-CGCGCGACCACGACACGAAA-BHQ-1	Weisenberger, D.J. et al Nature Genet **38**, 787-793 (2006).
**NEUROD2**	NEUROD2-M1B	HB-260	Neurogenic Differentiation 2	17q12	AC087491	Hs.322431	38463-38551	GGTTTGGTATAGAGGTTGGTATTTCGT	ACGAACGCCGACGTCTTC	6FAM-CGCCATACGAACCGCGAAACGAATATAA-BHQ-1	Weisenberger, D.J. et al Nature Genet **38**, 787-793 (2006).
**PSEN1**	PSEN1-M1B	HB-262	Presenilin 1 (Alzheimer Disease 3)	14q24.3	AF205592	Hs.592324	6663-6739	GTCGGGTGGAGAGAGATTTCG	AACACCTACGCCCTAAAACGTC	6FAM- TCGAACAAACAACATTTCCGAACCAAAACT-BHQ-1	Weisenberger, D.J. et al Nature Genet **38**, 787-793 (2006).
**HOXA1**	HOXA1-M2B	HB-268	Homeobox Gene A1	7p15	AC004079	Hs.67397	78138-78220	TTGTTTATTAGGAAGCGGTCGTC	TCGAACCATAAAATTACAACTTTCCA	6FAM-TCGTACGCGATCAACGCCAACAATTA-BHQ-1	Weisenberger, D.J. et al Nature Genet **38**, 787-793 (2006).
**CDKN2A**	CDKN2A-M3B	HB-269	Cyclin-dependent kinase inhibitor 2A; p16	9p21	AL449423	Hs.512599	61836-61933	GCGTTCGAGTGGCGGA	CTCCCGAACAACGTCGTACAC	6FAM-CAATTAAACTCCGCGCCGTAAAACAACAA-BHQ-1	GenBank Number: AL449423; Amplicon Location: 61836-61933.

TABLE S2- METHYLIGHT REACTION DETAILS
